# Proposition and application of an environmental salubrity index in rural agglomerations

**DOI:** 10.11606/s1518-8787.2022056003548

**Published:** 2022-05-18

**Authors:** Débora de Lima Braga, Nolan Ribeiro Bezerra, Paulo Sérgio Scalize

**Affiliations:** I Universidade Federal de Goiás Escola de Engenharia Civil e Ambiental Programa de Pós-Graduação em Engenharia Ambiental e Sanitária Goiânia GO Brasil Universidade Federal de Goiás. Escola de Engenharia Civil e Ambiental. Programa de Pós-Graduação em Engenharia Ambiental e Sanitária. Goiânia, GO, Brasil; II Instituto Federal de Educação. Ciência e Tecnologia de Goiás Goiânia GO Brasil Instituto Federal de Educação. Ciência e Tecnologia de Goiás. Goiânia, GO, Brasil

**Keywords:** Indicators (Statistics, Environmental Salubrity, Rural Areas, Social Planning, Environment and Public Health

## Abstract

**OBJECTIVE:**

Propose an *Índice de salubridade ambiental* (ISA_Rural _– environmental salubrity index) that expresses the conditions experienced in rural agglomerations, including indicators and subindicators for its subsequent application in rural communities in the state of Goiás.

**METHODS:**

We developed the research in three phases: 1) previous analysis for the proposition of an ISA_Rural_, with the participation of seven specialists; 2) proposition of the ISA_Rural_ by means of the Delphi method, starting with 168 specialists from 26 federative units of Brazil and Distrito Federal; and 3) application of the ISA_Rural_ in 43 rural communities in the state of Goiás.

**RESULTS:**

The proposed ISA_Rural_ resulted in the composition of eight indicators, four of which related to basic sanitation, and the others to health, socioeconomic conditions, public services offered, and housing conditions. The weight assigned to each indicator ranged from 22.82% for the water supply indicator to 6.35% for the service indicator, it is possible to apply the ISA_Rural_ fully or to evaluate each indicator individually. The application of ISA_Rural_ in communities of Goiás classified 86% of them with low salubrity, highlighting the worst conditions for *quilombola* communities. The sanitary sewage had the lowest score among the ISA_Rural_ indicators, requiring greater attention from public authorities.

**CONCLUSIONS:**

This study contributed to the proposition of an index in line with the concept of environmental salubrity, useful in the scope of public policies as a conditioner for the prioritization of actions needed to improve the salubrity conditions identified. The proposed ISA_Rural_ can be fully applied or used in the individual evaluation of each indicator of its composition. The results of its application made it possible to identify the communities with the worst environmental salubrity conditions and the indicators that require greater priority attention in the communities studied.

## INTRODUCTION

Health is the result of living conditions of a population, expressing the social and economic organization of the country, having as determinants and conditioning factors: food, housing, basic sanitation, environment, work, income, education, physical activity, transportation, leisure and access to essential goods and services, among others^1^.

Thus, these basic individual and collective needs promote the environmental salubrity of a population. Internationally, there is no direct concept of environmental salubrity, since the terminology is presented by the expression environmental health, and, in Brazil, differs from the concept of *salubridade ambiental* (environmental salubrity). In general, papers use the terms health, hygiene and cleaning to address the salubrious issue. In Brazil, environmental salubrity was initially defined by State Law no. 750, of March 31, 1992, in Article 2, Section II, “as the environmental quality capable of preventing the occurrence of diseases transmitted by the environment and of promoting the improvement of mesological conditions favorable to the health of the urban and rural population”^2^. This concept has been undergoing changes, as presented in several publications^3^.

The study of the environmental salubrity of a place is important to measure the health situation that a certain population enjoys as a result of their living conditions. Therefore, it is possible to measure a healthy environment by determining the health status of a population, influenced by socioeconomic conditions, education, basic sanitation, and the environments in which they circulate daily.

In this context, to determine environmental salubrity, the *Conselho Estadual de Saneamento* (Conesan – State Sanitation Council)^6^proposed the *indicador de salubridade ambiental* (ISA – Environmental Salubrity Indicator), from which its original composition has been adapted, with the inclusion and exclusion of indicators and/or subindicators and the alteration of their weights. Many times this occurs arbitrarily or through the replication of existing studies, considering, or not, the peculiarities of the analyzed region^7^. It is important to select carefully the indicators to compose the ISA, interrelating its problem and objective of analysis. Few studies have used the literature review^10^ and employed the Delphi method^11 , 12^to propose an index.

Despite the good acceptability of ISA, little research exists on environmental salubrity in rural areas. Of 76 studies on the ISA^9^, only seven were applied to rural areas, where only one study adapted the ISA, considering the conceptual relations of sanitation and health. However, the object of study in that case was the rural households, not the rural agglomeration^13^.

Thus, the objective of this work was to propose an index to determine environmental salubrity in rural agglomerations (ISA_Rural_) and apply it to rural communities in the state of Goiás.

## METHODS

We carried out the research methodology in three phases, preceded by a literature review using the following databases: Scientific Electronic Library Online (SciELO); *Periódicos* Capes; Web of Science, and other online search tools. For this, we used the keywords in English and Portuguese: “indicator”; “index”; “salubrity”; “environmental health”; “environmental”; “health”; “ *indicador* ”; “ *índice* ”; “ *salubridade* ”; “ *salubridade ambiental* ”; “ *saúde ambiental* ”; “ *indicador de salubridade* ” and “ISA”. The material found provided subsidies for the elaboration of the forms used in the first and second phases.

### Phase One: Preliminary Analysis to Propose an ISARural

We carried out this phase in order to define the methodology to apply for the proposition of an ISA_Rural_. For this, we selected specialists based on their area of expertise, related to ISA and to environmental indicators or environmental health, in addition to their availability to contribute to the project. Therefore, we chose seven experts who could participate in the activities and be present at a face-to-face activity. In order to guide and bring subsidies for the discussions, we prepared and applied a semi-structured interview form containing: the program, the purpose of ISA, concepts of environmental salubrity, the Basic Manual of ISA^6^, and six guiding questions (in a complementary file)^a^. After planning the answers, we had a meeting, in Goiânia, on March 20, 2019, when we discussed the topic, culminating with the indication of a method to apply in the proposition of an ISA_Rural_, besides the initial indicators useful for its composition and the definition of consultation with experts per domain area.

### Phase Two: ISARural Proposition

We built the ISA proposition using the Delphi method, useful to structure the communication process of a group in such a way that it can, in an integrated way, deal with complex problems^14^. We built it in the following sequence:

### Selection of Experts

- Group 1: made up of 168 specialists from all the Federal Units (UF) of Brazil, the Distrito Federal, and representatives of the rural communities, with their areas of expertise related to the research, who guided the choice and evaluation of the indicators for the ISA_Rural_.
- Group 2: made up of 66 members formed from the members of Group 1 who agreed to participate in the research; and two more researchers from the environmental health area. We subdivided them by areas of activity (water supply; sewage; solid waste; rainwater; environmental health; management; and community) and used them for the choice and evaluation of the subindicators.

a) 1st step: Indicator Selection
  - 1st round: choice of indicators pre-selected by the experts in the face-to-face discussion, as well as the suggestion of new indicators and subindicators for each proposed indicator. We used a form with the contextualization steps to choose the indicators and suggest subindicators.
  - 2nd round: reevaluation of the answers in light of the answers of the other experts and inclusion, or exclusion, of the indicators suggested in the 1st round. Suggestion and evaluation of new indicators by means of a form containing the results of the 1st round.
b) 2nd step: Evaluation of indicators
  - ISA_Rural _formulation: presentation of the chosen indicators and weighting of the ISA_Rural_ indicators by applying a form containing the results of the 1st and 2nd rounds.
c) 3rd step: Selection and evaluation of subindicators
  - 1st round: aimed at choosing and weighting of the subindicators and suggesting new ones. It counted on the application of forms with the results of the first round of choice of indicators sent to each subgroup of experts related to their area of work, by means of which occurred the presentation and analysis of the suggested subindicators. Then, we selected and evaluated the subindicators for each ISA_Rural_.
  - 2nd round: reevaluation of the responses of the other experts. By applying a form containing the results of the first round of analysis, we selected and evaluated the subindicators of each ISA_Rural_^b^.

The Research Ethics Committee of the Universidade Federal de Goiás (UFG) approved the project, with consultation with experts, under protocol no. 3.893.454/2020.

### Phase Three: Applying ISARural

The third and last phase consisted in applying the ISA_Rural_ and in measuring and analyzing the environmental salubrity in 43 rural and traditional communities in the state of Goiás, being 16 agglomerations, 21 *quilombolas* , and six riverside communities ( Table 3 ). The data for the calculation of the ISA came from the project sanitation and environmental health in rural and traditional communities of Goiás (SanRural), developed by UFG and financed by the National Health Foundation (Funasa), of which the authors are part. We collected the data locally, including water analysis, blood and stool tests, application of forms and checklists to survey the conditions of sanitation, health, housing, hygiene, soil use and occupation, collective infrastructure, and socioeconomic conditions. The Research Ethics Committee of the UF Goiás approved the project, under protocol no. 2.886.174/2018.

**Table 3:** Decreasing position and values of the ISARural indicators of rural communities in the state of Goiás classified according to their salubrity.

Community name and typology	I_AB_	I_ES_	I_MRS_	I_MAP_	I_Health_	I_SE_	I_Services_	I_CM_	ISA_Rural_
Julião Ribeiro^a^	64.55	65.92	32.59	75.27	82.99	36.50	55.70	85.69	**62.71**
Povoado Veríssimo^c^	77.23	5.26	65.18	48.30	74.88	50.37	98.48	80.51	**58.41**
Tarumã^a^	70.22	55.93	22.09	59.93	70.02	29.73	71.36	80.85	**57.81**
Monte Moriá^a^	60.67	48.09	19.70	52.97	71.47	37.70	56.66	75.68	**52.72**
Itajá II^a^	57.45	25.12	32.70	58.60	79.48	34.60	71.09	82.89	**51.97**
João de Deus^a^	75.10	0.00	33.07	60.07	78.97	27.13	84.72	83.54	**51.49**
Vazante^b^	42.12	18.91	64.24	61.01	65.28	36.92	84.72	82.63	**50.87**
Mesquita^b^	55.18	7.38	38.54	64.04	75.36	46.92	71.60	76.43	**49.20**
Extrema^b^	70.01	2.65	34.91	44.34	71.23	30.25	83.89	79.86	**48.77**
Povoado Vermelho^b^	64.88	19.52	18.72	62.36	69.14	22.96	80.83	73.92	**48.52**
Engenho da Pontinha^a^	64.88	0.00	31.85	55.56	74.97	19.03	84.72	91.42	**48.16**
Lageado^a^	64.88	4.23	24.16	48.51	67.98	23.96	100.00	79.97	**46.89**
Castelo, Retiro e Três Rios^b^	53.69	3.63	23.41	60.28	79.37	28.38	97.62	80.03	**46.71**
Fio Velasco^c^	46.84	37.64	46.24	28.72	64.30	14.34	56.66	74.44	**46.08**
Registro do Araguaia^c^	49.49	6.57	42.09	57.32	65.25	26.13	84.72	82.87	**46.03**
Fortaleza^a^	64.88	0.00	24.86	53.96	70.20	21.50	84.72	82.36	**45.90**
Santa Fé da Laguna^a^	56.46	1.81	24.70	69.48	68.08	31.48	83.89	79.46	**45.81**
Arraial das Pontes^c^	49.71	0.00	56.51	78.21	59.77	18.89	56.66	88.08	**45.71**
Forte^b^	64.88	0.00	17.57	57.72	70.50	32.70	84.72	70.97	**45.21**
Fazenda Santo Antônio da Laguna^b^	53.50	0.00	22.19	58.56	80.06	16.57	83.52	88.87	**44.64**
Queixo Dantas^b^	61.88	0.00	19.70	60.28	72.53	13.45	84.72	80.79	**44.48**
Landi^c^	69.28	0.00	19.70	48.66	70.08	23.65	56.66	84.80	**44.42**
Povoado Moinho^b^	40.61	6.80	33.56	60.08	75.13	28.44	84.72	78.78	**44.21**
Sumidouro^b^	51.93	2.44	30.84	57.55	75.37	26.74	56.66	84.75	**44.03**
Rochedo^a^	37.26	0.00	29.98	57.87	77.70	34.77	100.00	80.19	**43.44**
Céu Azul^a^	43.43	0.00	22.88	52.65	73.95	43.32	100.00	76.32	**43.43**
São Lourenço^a^	44.71	0.00	27.66	57.48	78.33	21.05	99.24	75.32	**43.20**
Piracanjuba^a^	42.12	0.00	37.52	47.25	76.01	31.73	84.72	79.33	**43.19**
Almeidas^b^	58.01	0.00	31.05	51.99	64.06	18.02	84.72	71.90	**42.96**
São Sebastião da Garganta^a^	42.04	0.00	29.62	64.73	66.55	33.46	84.72	80.81	**42.60**
Madre Cristina^a^	51.30	3.97	28.05	50.46	68.45	27.76	74.17	65.89	**41.87**
Olhos d’água^c^	49.14	0.00	19.70	50.74	79.33	4.92	84.72	84.33	**41.26**
Água Limpa^b^	51.30	0.00	24.35	51.87	75.44	23.11	55.76	76.12	**40.96**
Rafael Machado^b^	35.64	0.00	30.84	54.94	70.21	36.60	83.18	77.27	**40.84**
Taquarussu^b^	37.68	0.00	19.70	54.06	77.48	31.75	81.09	60.42	**38.59**
São Domingos^b^	49.99	3.53	19.15	60.26	76.53	18.08	64.13	39.13	**38.16**
Canabrava^b^	24.69	0.00	19.70	55.39	80.48	25.45	83.21	78.95	**37.39**
Quilombo do Magalhães^b^	36.10	0.00	16.89	59.75	73.29	6.27	53.80	84.33	**36.02**
José de Coleto^b^	32.76	0.00	17.51	65.81	63.96	24.48	83.12	42.40	**34.24**
Arraial da Antas^a^	34.80	0.00	24.16	52.76	69.20	6.27	100.00	37.27	**34.24**
Baco Pari^b^	7.02	2.44	20.09	50.01	72.92	14.18	84.72	59.69	**29.86**
Porto Leucádio^b^	8.42	0.00	19.70	56.96	74.59	20.52	56.66	67.14	**29.86**
Pelotas^b^	5.52	0.00	19.70	63.58	83.84	14.68	56.66	51.69	**28.96**
Mean	49.35	7.49	28.78	56.75	72.90	25.92	78.46	75.30	**44.23**

^a^ Agglomeration.

^b^ *Quilombola* .

^c^ Riverside.

Note: environmental salubrity conditions: blue = salubrity (from 76 to 100 points), green = medium salubrity (from 51 to 75 points), orange = low salubrity (from 26 to 50 points), and red = insalubrity (0 to 25 points).

We calculated all the indicators and subindicators that made up the ISA_Rural_ using Microsoft Excel software. We presented the results for each community studied, as well as from the worst to the best environmental salubrity condition among them, according to the following scoring ranges: insalubrious (between 0 and 25), low salubrity (from 26 to 50), medium salubrity (from 51 to 75) or salubrious (from 76 to 100)^3^.

## RESULTS AND DISCUSSION

### Preliminary Analysis for the Proposition of an ISARural

The meeting in person started with a discussion about the answers of 57.14% of the experts consulted in the first phase. Based on the existing concepts of environmental salubrity and having as main reference the concept currently used by Funasa^5^, we discussed and proposed, together with the specialists who contributed to the study, that “environmental salubrity consists of the health situation that a certain population enjoys as a result of the socioeconomic and environmental conditions in which they live”. We used this as a reference for the determination of indicators and subindicators and their weightings.

Due to the diversity of the rural environment, we defined the ISA_Rural_ proposition to be for rural agglomerations, and not for all rural areas. The Brazilian Institute of Geography and Statistics (IBGE) defines rural agglomerations as residential units with adjacent buildings, that is, 50 meters or less in distance from each other and with characteristics of permanence^15^. In this sense, ISA_Rural_ can be applied to census sectors:1b, 2 and 4 (agglomerations close to urban areas); 3 (more densely populated isolated agglomerations), 5, 6 and 7 (less densely populated isolated agglomerations), defined in the *programa nacional de saneamento rural* (PNSR – national rural sanitation program)^16^, one of the three programs of the *Plano Nacional de Saneamento Básico* (Plansab – National Basic Sanitation Plan)^17^.

By consensus of the experts, we chose Delphi as the most appropriate method, developed in three stages: 1) choice and/or complementation of the indicators suggested in the meeting; 2) evaluation of the indicators, and 3) choice and evaluation of the subindicators. Initially, we suggested seven indicators for the consultation with experts: *indicador de abastecimento de água* (I_AB_ – water supply indicator); *indicador de esgotamento sanitário* (I_ES_ – sewage indicator); *indicador de resíduos sólidos* (I_RS_ – solid waste indicator); *indicador de drenagem* (I_DR_ – drainage indicator); health indicator (I_Health_); *indicador socioeconômico* (I_SE_ – socioeconomic indicator); and service indicator (I_Services_). Finally, we defined that the specialists should be selected and divided by areas of expertise, composing seven groups, four related to basic sanitation, one to environmental health, and two others to environmental management and rural communities. The last two groups have the function of allowing the analysis of the composition of the indicators and revealing, by the representatives of the communities, the particularities and limitations of the rural areas. Thus, the previous analysis phase fulfilled the task of defining the methodology for the ISA_Rural_ proposition.

### ISARural Proposition

After the consensus obtained in the previous phase, we began proposing the ISA_Rural_ using the Delphi method, divided in three stages as described in Table 1 . It presents the number of invited specialists, the frequency and time of feedback, as well as the UF and the Distrito Federal without feedback, representing, at the end of the ISA_Rural_ composition, 70.4% of participation, which, given the geographical dimensions of the country, was considered excellent.

**Table 1:** Stages of the Delphi method application, with the number of invited experts, feedback frequency, period, and Brazilian federative units without feedback of experts.

Stages of the Delphi method		Number of invited experts	Feedback from the experts (%)	Period (days)	Participation of UF representatives (%)	UF without answer back
1ª	1st round for choosing and suggesting indicators and suggesting subindicators	168	38.1	52	85.2	MT, MS, PA and PE
	2nd round for choosing the indicators	64	84.4	46	77.8	MT, MS, PA, PE, AM and RS
2ª	Weighting of indicators	54	87.0	37	70.4	MT, MS, PA, PE, AM, RS, ES and SE
3ª	1st round for analysis, choice and weighting of subindicators	66	60.6	60	74.1	MT, MS, PA, PE, AM, AL and ES
	2nd round for analysis and weighting of subindicators	40	87.5	40	70.4	MT, MS, PA, PE, AM, AL, ES and RS

UF: Brazilian Federative Unit; AL: Alagoas; AM: Amazonas; ES: Espírito Santo; MT: Mato Grosso; MS: Mato Grosso do Sul; PA: Pará; PE: Pernambuco; RS: Rio Grande do Sul; SE: Sergipe.

**Figure 1:**
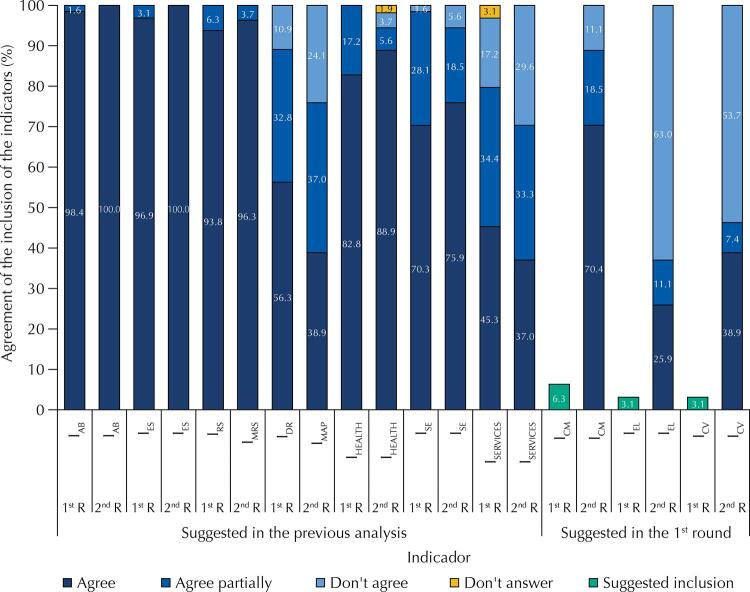
Frequency of agreement on the permanence and inclusion of indicators in the ISARural composition obtained in the first and second rounds of the first stage of the Delphi method.

The frequency of agreement of the seven indicators, defined in the previous analysis and suggested in the 1st and 2nd rounds of selection of ISA_Rural_ indicators ( Table 1 ), is presented in Figure 1 , together with the frequency of suggestion of three new indicators suggested in the 1st round and the percentage of agreement of their inclusion. In the 1st round, the indicators I_AB_, I_ES_, I_RS_ and I_Health_ obtained 100% frequency of agreement, with only a few exceptions of partial agreement, such as the inclusion of the word “management” in the I_RS_, changing to *indicador de manejo de resíduos sólidos* (I_MRS_ – solid waste management indicator). Most experts agreed, totally or partially, with the I_DR_ (89.06%), I_SE_ (98.44%) and I_Services_ (79.69%).

I_AB_ ( *indicador de abastecimento de água* ): water supply indicator; I_ES_ ( *indicador de esgotamento sanitário* ): sewage indicator; I_RS_ ( *indicador de resíduos sólidos* ): solid waste indicator; I_MRS_ ( *indicador de manejo de resíduos sólidos* ): solid waste management indicator; I_DR_ ( *indicador de drenagem* ): drainage indicator; I_MAP_ ( *indicador de manejo de águas pluviais* ): rainwater management indicator; I_Health_: health indicator; I_SE_ ( *indicador socioeconômico* ): socioeconomic indicator; I_Services_: services indicator; I_CM_ ( *indicador de condições de moradia* ): housing conditions indicator; I_EL_ ( *indicador de energia elétrica* ): electrical power indicator; I_CV_ ( *indicador de controle de vetores* ): vector control indicator ; 1st R: first round; 2nd R: second round.

Regarding the I_DR_, the specialists who did not agree with its inclusion (10.94%) justified that its relevance is only for urban areas, because, for rural areas, drainage is a natural process, and that Federal Law no. 11.455/2007^18^only contemplates urban areas. However, we considered rainwater management in the PNSR^16^ indicators. For this indicator, the suggestion is to include the word “management”, with reference to the rural sanitation components of the PNSR, changing it to *indicador de manejo de águas pluviais* (I_MAP_ – rainwater management indicator) in place of the I_DR_. Regarding the I_SE_, only one expert did not agree with its inclusion, but did not provide any justification. For I_Services_, the reasons for not agreeing were that it is a very broad indicator, it is difficult to obtain data, and it is included in the previous indicators. In this round, we observed the suggestion of 21 more indicators different from the initial seven, and we considered relevant the one suggested by two or more experts, resulting in three indicators: 1) *indicador de condições de moradia* (I_CM _– housing conditions indicator); 2) *indicador de energia elétrica* (I_EL _– electric power indicator); and 3) *indicador de controle de vetores* (I_CV _– vector control indicator) ( Figure 1 ).

Also during the 1st round, we separated the subindicators suggested by the specialists into groups that encompassed the same theme. We used those with higher frequency in the proposition of subindicators in the 3rd stage of the Delphi method application.

In the 2nd round, we presented all the questions and observations to the experts, with the option of performing a new analysis on the ISA_Rural_ composition. Among the ten suggested indicators, eight presented a frequency greater than 70% of agreement (total and partial), which we maintained and considered for the weighting of the formula. We considered that the other indicators contemplated I_EL_ and I_CV_, and we removed them from the index. Eight indicators defined the ISA_Rural_, four related to basic sanitation components, one to health, one to socioeconomic conditions, one to services offered in rural agglomerations, and one to housing conditions. Thus, the following indicators remained: I_AB_; I_ES_; I_MRS_; I_MAP_; I_Health_; I_SE_; I_Services_; and I_CM_. The sanitation and health indicators accounted for 75.81% of the weight of the ISA_Rural_.

In the next round, relative to the 2nd stage of the Delphi method application, the experts considered the weights for each one of the indicators, resulting in the following average values and standard deviations for each indicator: I_AB_ = 22.82 ± 7.45; I_ES_ = 19.44 ± 5.29; I_MRS_ = 13.16 ± 4.01; I_MAP_ = 7.82 ± 3.39; I_Health_ = 12.55 ± 4.85; I_SE_ = 8.70 ± 3.92; I_Services_ = 6.35 ± 2.94 and I_CM_ = 9.16 ± 4.62. ISA_Rural_ resulted from the average value of the weights assigned by the experts for each indicator, resulting in Equation 1,


$${ISA}_{\text{Rural }}=0.2282I_{AB}+0.1944I_{ES}+0.1316I_{MRS}+0.0782I_{MAP}+0.1255I_{\text{Health }}+0.0870I_{SE}+0.0635I_{\text{Services }}+0.0916I_{CM}$$ (1)

Legend: water supply indicator = I_AB_; sewage indicator = I_ES_; solid waste management indicator = I_MR__S_; rainwater management indicator = I_MAP_; health indicator = I_HEALTH_; socioeconomic indicator = I_SE_; service indicator = I_Services_; and housing conditions indicator = I_CM_.

**Figure 2:**
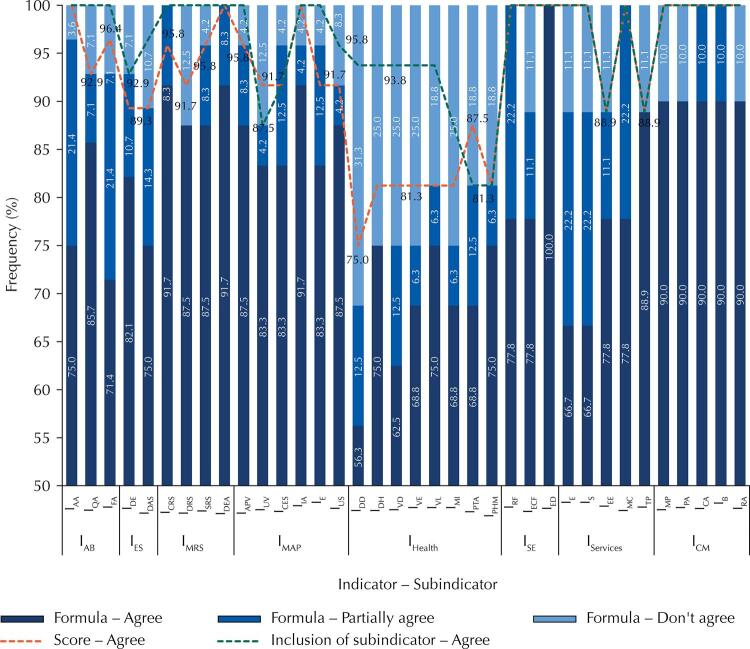
Agreement frequency on the inclusion of the subindicator for each indicator, as well as the suggested formulas and scores in the first round

For the 1st round of the 3rd stage of the Delphi method application, and based on the groups of subindicators that obtained the highest percentage of suggestion in the 1st round of the 1st stage, we consulted the specific technical-scientific bibliography, considering the concept of environmental salubrity, used to formulated a list of subindicators sent for consultation to the specialists. Figure 2 shows the frequency of agreement of the inclusion of subindicators in the formulas and the scores.

I_AB_ ( *indicador de abastecimento de água* ): water supply indicator; I_ES_ ( *indicador de esgotamento sanitário* ): sewage indicator; I_MRS_ ( *indicador de manejo de resíduos sólidos* ): solid waste management indicator; I_MAP_ ( *indicador de manejo de águas pluviais* ): rainwater management indicator; I_Health_: health indicator; I_SE_ ( *indicador socioeconômico* ): socioeconomic indicator; I_Services_: services indicator; I_CM _( *indicador de condições de moradia* ): housing conditions indicator; I_AA_ ( *subindicador de abastecimento adequado de água no domicílio* ): adequate domestic water supply subindicator; I_QA_ ( *subindicador de qualidade da água* ): water quality subindicator; I_FA_ ( *subindicador de frequência no abastecimento de água* ): frequency in water supply subindicator; I_DE_ ( *subindicador de destinação adequada de excretas* ): adequate disposal of excreta subindicator; I_DAS _( *subindicador de destinação adequada de águas servidas* ): adequate disposal of wastewater subindicator; I_CRS_ ( *subindicador de coleta adequada de resíduos sólidos* ): adequate solid waste collection subindicator; I_DRS_ ( *subindicador de destinação adequada de resíduos sólidos* ): adequate disposal of solid waste subindicator; I_SRS_ ( *subindicador de separação dos resíduos sólidos* ): solid waste separation subindicator; I_DEA_ ( *subindicador de destinação adequada de embalagens de agrotóxicos* ): adequate disposal of agrochemical packaging subindicator; I_APV_ ( *subindicador de manejo de águas pluviais adequados nas vias* ): adequate rainwater management on roads subindicator; I_UV_ ( *subindicador de dificuldade ou impossibilidade de utilização das vias de acesso* ): difficulty or impossibility to use the access routes subindicator; I_CES_ ( *subindicador de controle de escoamento superficial* ): runoff control subindicator; I_IA_ ( *subindicador de ocorrência de inundação e alagamento* ): flooding and inundation occurrence subindicator; I_E_ ( *subindicador de erosões* ): erosion subindicator; I_US_ ( *subindicador de uso do solo* ): soil use subindicator; I_DD_ ( *subindicador de ocorrência de diarreia* ): diarrhea occurrence subindicator; I_DH_ ( *subindicador de ocorrência de hepatite A* ): hepatitis A occurrence subindicator; I_VD_ ( *subindicador de dengue* ): dengue subindicator ; I_VE_ ( *subindicador de esquistossomose* ): schistosomiasis subindicator; I_VL_ ( *subindicador de leptospirose* ): leptospirosis subindicator ; I_MI_ ( *subindicador de mortalidade infantil* ): infant mortality subindicator; I_PTA_ ( *subindicador de tratamento da água domiciliar* ): household water treatment subindicator; I_PHM_ ( *subindicador de higienização das mãos* ): hand hygiene subindicator; I_RF_ ( *subindicador de renda per capita familiar* ): *per capita* household income subindicator; I_ECF_ ( *subindicador de escolaridade do chefe de família* ): education of the head of household subindicator; I_ED_ ( *subindicador de educação* ): education subindicator ; I_E_ ( *subindicador de educação* ): education subindicator; I_S_: ( *subindicador de saúde* ): health subindicator; I_EE_ ( *subindicador de energia elétrica* ): electric power subindicator; I_MC_ ( *subindicador de meio de comunicação* ): communication means subindicator; I_TP_ ( *subindicador de transporte público* ): public transportation subindicator; I_MP_ ( *subindicador de material usado na parede* ): wall material subindicator; I_PA_ ( *subindicador de piso adequado* ): adequate flooring subindicator; I_CA_ ( *subindicador de cobertura adequada* ): adequate coverage subindicator; I_B_ ( *subindicador de banheiro* ): bathroom subindicator; I_RA_ ( *subindicador de reservação interna adequada de água* ): adequate internal water reservoir subindicator.

The subindicators of I_AB_, I_MRS_, I_SE_ and I_CM_ obtained 100% frequency of agreement for inclusion, with some reservations of adjustments in the descriptions of the formulas and weightings.

In the I_ES_ subindicators, only two experts (7.1%) did not agree with the inclusion, justifying that it would not be necessary to separate sanitary sewage into excreta and wastewater. However, studies applied in rural areas^13^ considered this separation relevant. Therefore, we kept these subindicators for the next round, with only minor changes in the formulas and scores, according to the suggestions.

Regarding I_MAP_, half of the subindicators (I_APV_, I_IA_ and I_E_) obtained 100% agreement, and the other half obtained 87.5% frequency of agreement for I_UV_, 91.7% for I_CES_ and 95.8% for I_US_. The justification was the irrelevance of these indicators, also influencing the answers obtained in the formulas and scores.

As for I_Services_, only the subindicators I_EE_ and I_TP_ did not obtain inclusion agreement in 100%, with 88.9%. In the descriptions of the formulas, the disagreement (11.1%) occurred in the I_E_, I_S_, I_EE_, and I_TP_ subindicators, among which the suggestion was that the criterion of service attendance was included in the I_E_ and I_S_ subindicators.

The subindicators of I_Health_, despite having an inclusion concordance of more than 80%, presented several considerations in the formula descriptions. One of them was the modification of the sampling form, from household to inhabitants, obtaining the occurrence of the disease. Because it changes the whole calculation form, we presented the changes suggested for evaluation in the 2nd round of the 3rd stage to the experts. For the other indicators, we present only the subindicator weighting option.

In the last round, we weighted all the subindicators with the average of the assigned weights and also obtained the frequency of agreement of the changes in the formulas of the I_Health_ subindicators. Only one expert disagreed with the home water treatment subindicator (I_PTA_); the others fully agreed. Table 2 shows the final formulas for the indicators and their respective subindicators and scores.

**Table 2:** Formulas for calculating indicators IAB, IES, IMRS, IMAP, IHealth, ISE, IServices and ICM, that make up the ISARural and its subindicators, with description and scoring.

$I_{AB}=0.4212I_{AA}+0.3512I_{QA}+0.2277I_{FA}$			
Subindicator	Formula	Score	Description
Adequate water supply at home (I_AA_)	$I_{AA}=\frac{\text{ Draa }}{Drt}\times 100\%$	By formula	D_raa_ = number of households in the rural agglomeration supplied by a water distribution network, with indoor plumbing at the residence or on the property, or by a well, water source, or rainwater collection cistern, with indoor plumbing D_rt_ = number of households in the rural agglomeration
Water quality (I_QA_)	$I_{QA}=\frac{NAA}{NRA}\times 100\%$	$\begin{array}{c} I_{QA}=100\%-\text{ Score }=100\\ I_{QA}=95\text{ to }100\%-\text{ Score }=80\\ I_{QA}=85\text{ to }95\%-\text{ Score }=60\\ I_{QA}=70\text{ to }85\%-\text{ Score }=40\\ I_{QA}=50\text{ to }70\%-\text{ Score }=20\\ I_{QA}<50\%-\text{ Score }=0 \end{array}$	N_AA_ = quantity of samples in accordance with acceptable water quality values for colimetry, chlorine and turbidity N_RA_ = number of samples performed
Frequency of water supply (I_FA_)	$I_{FA}=\frac{\mathrm{Drfa}}{Drt}\times 100\%$	By formula	D_rfa_ = number of rural households that never or rarely lack water (1 time per month) D_rt_ = number of households in the rural agglomeration
$I_{ES}=0.6349I_{DE}+0.3651I_{DAS}$			
**Subindicator**	**Formula**	**Score**	**Description**
Adequate disposal of excreta (I_DE_)	$I_{DE}=\frac{\text{ Dre }}{\text{ Drt }}\times 100\%$	By formula	D_re_ = number of households in the rural agglomeration served by a collecting system followed by treatment, septic tank or sewage treatment technologies in the rural area for excreta D_rt_ = number of households in the rural agglomeration
Adequate disposal of wastewater (I_DAS_)	$I_{DAS}=\frac{\text{ Dras }}{\text{ Drt }}\times 100\%$	By formula	D_ras_ = number of households in the rural agglomeration served by a collecting system followed by treatment, septic tank or rural sewage treatment technologies for wastewater D_rt_ = number of households in the rural agglomeration
$I_{\text{MRS }}=0.2817I_{\text{CRS }}+0.2985I_{\text{DRS }}+0.1970I_{\text{SRS }}+0.2228I_{DEA}$			
**Subindicator**	**Formula**	**Score**	**Description**
Adequate collection of solid waste (I_CRS_)	$I_{CRS}=\frac{Drc}{Drt}\times 100\%$	By formula	D_rc_ = number of households in the rural agglomeration served by direct or indirect solid waste collection systems with a frequency of at least once a week D_rt_ = number of households in the rural agglomeration
Adequate disposal of solid waste (I_DRS_)	$I_{DRS}=\left(1-\frac{Drd}{Drt}\right)\times 100\%$	By formula	D_rd_ = number of households in the rural agglomeration that bury, burn or dispose openly solid waste D_rt_ = number of households in the rural agglomeration
Separation of solid waste (I_SRS_)	$I_{SRS}=\frac{DrS}{Drt}\times 100\%$	By formula	D_rs_ = number of households in the rural agglomeration that separate their solid waste D_rt_ = number of households in the rural agglomeration
Proper disposal of pesticide packaging (I_DEA_)	$I_{DEA}=\frac{\text{ Drea }}{\text{ Drta }}\times 100\%$	By formula	D_rea_ = number of households in the rural agglomeration that return their pesticide packages to the producer, the seller of the product, or to a voluntary delivery point D_rta_ = number of households in the rural agglomeration using pesticides
$I_{MAP}=0.1639I_{APV}+0.1308I_{UV}+0.1580I_{CES}+0.2133I_{IA}+0.1721I_{E}+0.1619I_{US}$			
**Subindicator**	**Formula**	**Score**	**Description**
Adequate rainwater management on roads (I_APV_)	$I_{APV}=\frac{Drvp}{Drt}\times 100\%$	By formula	D_rvp_ = number of households in the rural agglomeration located on roads with pavement, curbs, and manholes D_rt_ = number of households in the rural agglomeration
Difficult or impossible to use access roads (I_UV_)	$I_{UV}=\frac{Drac}{Drt}\times 100\%$	By formula	D_rac_ = number of households in the rural agglomeration that did not experience access difficulties to their homes in the last five years D_rt_ = number of households in the rural agglomeration
Control of surface runoff (I_CES_)	$I_{CES}=\frac{\text{ Drce }}{Drt}\times 100\%$	By formula	D_rce_ = number of households in the rural agglomeration with excess runoff control devices, such as contour lines, channels or ditches, or others D_rt_ = number of households in the rural agglomeration
Occurrence of flooding and inundation (I_IA_)	$I_{IA}=\frac{\text{ Dria }}{Drt}\times 100\%$	By formula	D_ria_ = number of households in the rural agglomeration without flooding in the last five years and inundation D_rt_ = number of households in the rural agglomeration
Erosions (I_E_)	$I_{E}=\frac{\text{ Dre }}{Drt}\times 100\%$	By formula	D_re_ = number of properties in the rural agglomeration that did not show erosion D_rt_ = number of households in the rural agglomeration
Soil use (I_US_)	$I_{US}=\mathrm{CuS}\times 100\%$	By formula	Predominant soil use of rural agglomeration (C_us_), criteria: native vegetation: 1; pasture: 0.5; agriculture: 0.25; exposed soil: 0
$I_{\text{Health }}=0,1557I_{DD}+0,1292I_{DH}+0,1038I_{VD}+0,1018I_{VE}+0,0941I_{VL}+0,1710I_{MI}+0,1414I_{PTA}+0,1030I_{PHM}$			
**Subindicator**	**Formula**	**Score**	**Description**
Diarrhea occurrence (I_DD_)	$I_{DD}=\left(1-\frac{Hrdd}{Hrt}\right)\times 100\%$	By formula	H_rdd_ = number of inhabitants living in the rural agglomeration with diarrhea in the last month. H_rt_ = number of inhabitants living in the rural agglomeration
Hepatitis A Occurrence (I_DH_)	$I_{DH}=\left(1-\frac{Hrdh}{Hrt}\right)\times 100\%$	By formula	H_rdh_ = number of inhabitants living in the rural agglomeration diagnosed with hepatitis A H_rt_ = number of inhabitants living in the rural agglomeration
Dengue (I_VD_)	$I_{VD}=\left(1-\frac{Hrvd}{Hrt}\right)\times 100\%$	By formula	H_rvd_ = number of residents in the rural agglomeration diagnosed with dengue, zika, chikungunya or yellow fever H_rt_ = number of inhabitants living in the rural agglomeration
Schistosomiasis (I_VE_)	$I_{VE}=\left(1-\frac{\text{ Hrve }}{Hrt}\right)\times 100\%$	By formula	H_rve_ = Number of inhabitants living in the rural agglomeration diagnosed with schistosomiasis H_rt_ = number of inhabitants living in the rural agglomeration
Leptospirosis (I_VL_)	$I_{VL}=\left(1-\frac{Hrvl}{Hrt}\right)\times 100\%$	By formula	H_rvl_ = number of inhabitants living in the rural agglomeration diagnosed with leptospirosis H_rt_ = number of inhabitants living in the rural agglomeration
Infant Mortality (I_MI_)	$I_{MI}=\left(1-\frac{Crmi}{Crt}\right)\times 100\%$	By formula	C_rmi_ = number of children under 1 year old living in the rural agglomeration with death in the last year C_rt_ = total number of children under 1 year old residing in the rural agglomeration
Domestic water treatment (I_PTA_)	$I_{PTA}=\frac{Drta}{Drt}\times 100\%$	By formula	D_rta_ = number of households in the rural agglomeration performing some treatment on their drinking water, such as filtration, boiling or disinfection D_rt_ = number of households in the rural agglomeration
Hand hygiene (I_PHM_)	$I_{PHM}=\frac{\frac{Hrmr}{Hrt}+\frac{Hrmb}{Hrt}}{2}\times 100\%$	By formula	H_rmr_ = number of residents in the rural agglomeration who always wash their hands with soap and water before meals H_rmb_ = number of inhabitants living in the rural agglomeration who always wash their hands with soap and water after using the toilet H_rt_ = number of inhabitants living in the rural agglomeration
$I_{SE}=0,4389I_{RF}+0,2556I_{ECF}+0,3056I_{ED}$			
**Subindicator**	**Formula**	**Score**	**Description**
*Per capita* family income (I_RF_)	$I_{RF}=\frac{\mathrm{Drrf}}{Drt}\times 100\%$	By formula	D_rrf_ = number of households in the rural agglomeration with monthly per capita family income greater than or equal to one minimum wage D_rt_ = number of households in the rural agglomeration
Education of the head of household (I_ECF_)	$I_{ECF}=\frac{\text{ Drecf }}{\text{ Drt }}\times 100\%$	By formula	D_recf_ = number of households in the rural agglomeration whose head of household has at least completed elementary school D_rt_ = number of households in the rural agglomeration
Education (I_ED_)	$I_{ED}=\sqrt[{4}]{Epa*Fpj^{2}}$	By formula	Schooling of the adult population (E_pa_) = percentage of the rural agglomeration’s inhabitants aged 18 years or more with complete elementary education School attendance rate of the young population (F_pj_): arithmetic mean (1) of the percentage of children between 5 and 6 years old attending school; (2) of the percentage of young people between 11 and 13 years old attending the final years of regular elementary school; (3) of the percentage of young people between 15 and 17 years old with complete elementary school, and (4) of the percentage of young people between 18 and 20 years old with complete high school
$I_{\text{services }}=0,2222I_{E}+0,2806I_{S}+0,2000I_{EE}+0,1444I_{MC}+0,1528I_{TP}$			
**Subindicator**	**Formula**	**Score**	**Description**
Education (I_E_)	$I_{E}=E\times 100\%$	By formula	Basic education in the rural agglomeration (E), criterion: rural agglomeration is served by basic education service (school in the rural agglomeration or availability of school transport to a basic education unit) = 1; rural agglomeration is not served by public education service = 0
Health (I_S_)	$I_{S}=Sx100\%$	By formula	Health in the rural agglomeration (S), criterion: rural agglomeration is served by a health service (health center or community health workers) = 1; rural agglomeration is not served by a public health service = 0
Electric power (I_EE_)	$I_{EE}=\frac{\text{ Dree }}{\text{ Drt }}\times 100\%$	By formula	D_ree_ = number of households in the rural agglomeration with electric power. D_rt_ = number of households in the rural agglomeration
Means of communication (I_MC_)	$I_{MC}=\frac{Drmc}{Drt}\times 100\%$	By formula	D_rmc_ = number of rural agglomeration households with access to telephone, radio, television or internet D_rt_ = number of households in the rural agglomeration
Public Transportation (I_TP_)	$I_{TP}=Tp\times 100\%$	By formula	Public transport in the rural agglomeration (T_p_), criterion: rural agglomeration is served by public transport service = 1; rural agglomeration is not served by public transport service = 0
$I_{CM}=0,1430Imp+0,1505I_{PA}+0,1555I_{CA}+0,3125I_{B}+0,2385I_{RA}$			
**Subindicator**	**Formula**	**Score**	**Description**
Material used on the wall (I_MP_)	$I_{MP}=\frac{Drmp}{Drt}\times 100\%$	By formula	D_rmp_ = number of households in the rural agglomeration with masonry and plaster walls D_rt_ = number of households in the rural agglomeration
Adequate flooring (I_PA_)	$I_{PA}=\frac{\mathrm{Drpa}}{\mathrm{Drt}}\times 100\%$	By formula	D_rpa_ = number of households in the rural agglomeration with an impermeable floor or one that facilitates adequate cleaning D_rt_ = number of households in the rural agglomeration
Adequate Coverage (I_CA_)	$I_{CA}=\frac{\mathrm{Drca}}{Drt}\times 100\%$	By formula	D_rca_ = number of households in the rural agglomeration with tile roofing or other adequate rainwater insulation and thermal insulation D_rt_ = number of households in the rural agglomeration
Bathroom (I_B_)	$I_{B}=\frac{Drb}{Drt}\times 100\%$	By formula	D_rb_ = number of households in the rural agglomeration that have a bathroom with toilet and shower D_rt_ = number of households in the rural agglomeration
Adequate Internal Water Reservation (I_RA_)	$I_{RA}=\frac{\text{ Drra }}{\text{ Drt }}\times 100\%$	By formula	D_rra_ = number of households in the rural agglomeration with an internal water reservoir (water tank) that is capped and sanitized every six months mD_rt_ = number of households in the rural agglomeration with an internal reservoir

When comparing ISA_Rural_’s final proposition with studies found in the specific bibliography, we found that none of them contemplates, in an integral way, all the indicators. The separation of the specialists by area of expertise brought the formulation of essential subindicators with specificities, requiring easily obtainable data for calculation. Public authorities require some of them by means of PNSR^16^, and it is possible to obtain the others using questionnaires applied and used by the community health agents, improving them, as suggested in Bernardes, Bernardes and Gunther^13^.

### ISARural application

The application of the proposed ISA_Rural_ has found that only 14% of the communities are of medium salubrity, with the agglomerations occupying five of the top six places. In the remaining communities (86%), there is low salubrity ( Table 3 ), with 61.9% of the *quilombola* communities below average. Table 3 presents the decreasing position of the rural communities of Goiás, according to the results of the ISA_Rural _and its indicators.

Analyzing the I_AB_ separately, only the community Povoado Veríssimo (77.23%) received the classification of salubrious, and 48.84% of the communities received the classification of medium salubrity. The others, 39.5%, presented low salubrity conditions and 9.3% insalubrity. In the PNSR^16^ diagnostic, for the less densely populated isolated agglomerations, 46.3% of the inhabitants are adequately served in the water supply component, being close to the average value of 49.35 points ( Table 3 ) obtained for I_AB_. The low salubrity occurred mainly due to the quality of the water supply, with the presence of *E. coli* in most of the water samples analyzed, resulting in disagreement with Annex XX of Consolidation Ordinance no. 5 of the Ministry of Health^19^. The presence of *E. coli* in the water consumed by the population in rural communities has been reported in national and international scientific papers^20 , 21^, being something recurrent that requires attention from the public authorities. In most situations, disinfecting the water indoors with sodium hypochlorite solution would considerably decrease contamination^22^ and consequently improve healthiness. Among the indicators that make up the ISA_Rural_, the I_ES_ presented the worst results, present in 90.7% of the communities in unhealthy situations, requiring greater attention from the public authorities. This condition results from the use, in the vast majority of households, of a rudimentary cesspool as a solution for sanitary sewage. This result confirms the data presented in the PNSR, that only 15.2% of the inhabitants dispose of their effluents properly^16^, and the study by Roland et al.^23^ A study conducted in riverside communities in Amazônia concluded that one of the characteristics that most contribute to the situation of insalubrity and low salubrity is the precariousness of the houses in relation to the adequate disposal of excreta and grey waters^13^. Only two communities (4.65%) received the classification of medium salubrity, and another two (4.65%) as low salubrity.

Another worrisome basic sanitation component is solid waste management, represented by I_MRS_, present in only 6.98% of the communities served, in more than 80% of the households by direct or indirect solid waste collection. Although the great majority of the households in the communities separate their waste, they do not have adequate disposal, and burning is the main form of disposal, similar to the situation presented in the PNSR^16^ diagnostic and other studies^24^. The article 47 of the National Solid Waste Policy^25^forbid this practice. Depending on the composition of the waste, it can release toxic gases, and does not reduce all types of waste, contributing to the proliferation of diseases and influencing the quality of life of the population^23^. In view of the above about the I_MRS_, 53.49% of the communities fit as insalubrious, 39.53% with low salubrity and 6.98% medium salubrity.

In relation to the I_MAP_, we classified only the riverside community Arraial da Ponte, representing 2.33% of the analyzed communities, as salubrious. The presence of pavement, curbs, and manholes (a device that allows rainwater drainage) characterized this condition, serving 50% of the community. We classified the others, 76.74% as medium salubrity, and 20.93% as low salubrity. Rainwater management is the only sanitation component for which it was not possible to diagnose the current situation in rural areas of Brazil by PNSR^16^, because IBGE^26^ does not have enough data for such an analysis. For this reason, it is one of the biggest barriers to conducting studies on this component of basic sanitation, which stops the proper direction of public policies to solve problems related to infrastructure^23^.

I_Health_ was the third indicator to present the best results in the survey. We verified the salubrious situation registered in 30.2% of the communities and medium salubrious in 69.8%. This is mainly because the inhabitants of the communities have not been diagnosed by a health professional with schistosomiasis and/or leptospirosis, with the exception of one inhabitant of the Julião Ribeiro community, and no deaths of children under one year of age have occurred in these communities. However, many residents of the communities tested positive for hepatitis A, corroborating another study on rural agglomerations in the southwest of Goiás in which 82.20% of the residents had antibodies to the virus^27^, the main factor in the decrease in salubrity in this indicator.

The I_SE_ was the second indicator to present the worst salubrity results. Thus, 48.84% of the communities presented an insalubrity situation and 51.16% presented low salubrity due to the low education and monthly *per capita* income of the inhabitants. This consolidated the data presented in the PNSR^16^ and the analyses that the lower the levels of education and income, the worse the solutions adopted in basic sanitation^28^.

In general, I_Services_ showed the best results, with salubrity of 65.12% of the communities and 34.88% with medium salubrity. This is because 100% of the communities have basic education services, 69.77% have health services, and, in more than 90% of the households, 93% and 62.8% have access, respectively, to electricity and means of communication. The *Programa Nacional de Universalização do Acesso e Uso da Energia Elétrica* ^29^(National Program for the Universalization of Access to and Use of Electric Energy), responsible for the evolution of the universalization of access to energy, with a deadline of 2022, was extended several times. Therefore, it produces, and certainly will produce, improvements in social and economic dynamics for the communities not yet fully served by this fundamental service^30^.

Finally, the I_CM_ was the second indicator to show the best salubrity results, with 67.44% of the communities in a salubrious situation, 25.58% with medium salubrity, and 6.98% with low salubrity. In general, the communities have houses with adequate walls, floors and roofs, including the bathroom. However, their water reservoirs are in inadequate conditions, which may be one of the factors contributing to the low quality of the water and for being places of contamination^31^.

## CONCLUSIONS

The proposed ISA_Rural_ is in line with the concept of environmental salubrity. It is useful in the context of public policies, as a conditioner for the prioritization of actions necessary to improve the salubrity conditions in rural agglomerations, aiming to contribute to the health level of their populations. In addition, it allows an evaluation of the evolution of the goals in the PNSR and the Municipal Sanitation Plan. It is possible to apply this index in its totality or in the evaluation of each indicator that composes it.

The results of the application of ISA_Rural_ in the communities studied in the state of Goiás indicate that the public authorities should devote priority attention to implement actions aimed at the universalization of sanitary sewerage, followed by the improvement of socioeconomic conditions, particularly in *quilombola* communities, which presented the worst environmental salubrity conditions among the communities studied.

## Footnotes

a Supplementary material available from: https://files.cercomp.ufg.br/weby/up/780/o/Arquivo_complementar_proposicao_e_aplicacao_ISA_Rural.pdf

b Only the form referring to the I_Health_subindicators obtained one more stage for presentation and analysis of the changes suggested by the experts.
