# Supplementary material for: Proposition and application of an environmental salubrity index in rural agglomerations
Source: Rev Saude Publica. 2022 May 18;56:44. doi: 10.11606/s1518-8787.2022056003548 (PMC9165634; doi:10.11606/s1518-8787.2022056003548)
Supplement: Translation

# Translation: Proposição e aplicação de um índice de salubridade ambiental em aglomerados rurais

**Authors:** Débora de Lima Braga, Nolan Ribeiro Bezerra, Paulo Sérgio Scalize

**Affiliations:** I Brasil Universidade Federal de Goiás. Escola de Engenharia Civil e Ambiental. Programa de Pós-Graduação em Engenharia Ambiental e Sanitária. Goiânia, GO, Brasil; II Brasil Instituto Federal de Educação. Ciência e Tecnologia de Goiás. Goiânia, GO, Brasil

**Keywords:** Indicadores (Estatística, Salubridade Ambiental, Zona Rural, Planejamento Social, Meio Ambiente e Saúde Pública

## Abstract

**OBJETIVO:**

Propor um índice de salubridade ambiental que expresse as condições vividas em aglomerados rurais (ISA_Rural_), englobando indicadores e subindicadores para sua posterior aplicação em comunidades rurais do estado de Goiás.

**MÉTODOS:**

A pesquisa foi desenvolvida em três fases: 1) análise prévia para proposição de um ISA_Rural_, contando com a participação de sete especialistas; 2) proposição do ISA_Rural_por meio do método Delphi, iniciando com 168 especialistas das 26 unidades federativas do Brasil e do Distrito Federal; e 3) aplicação do ISA_Rural_em 43 comunidades rurais do estado de Goiás.

**RESULTADOS:**

O ISA_Rural_proposto resultou na composição de oito indicadores, sendo quatro relacionados ao saneamento básico, e os demais à saúde, às condições socioeconômicas, aos serviços públicos ofertados e às condições de moradia. O peso atribuído para cada indicador variou de 22,82%, para indicador de abastecimento de água, a 6,35%, para o indicador de serviços, podendo o ISA_Rural_ser aplicado na sua totalidade ou para avaliação de cada indicador individualmente. A aplicação do ISA_Rural_em comunidades de Goiás evidenciou que 86% se classificam com baixa salubridade, destacando as piores condições para as comunidades quilombolas. Dentre os indicadores do ISA_Rural_, o de esgotamento sanitário foi caracterizado com a menor pontuação, o que demanda uma maior atenção do poder público.

**CONCLUSÕES:**

Esse estudo cumpriu o papel de contribuir com a proposição de um índice em consonância com o conceito de salubridade ambiental, podendo ser empregado no âmbito das políticas públicas como um condicionante para a priorização das ações necessárias à melhoria das condições de salubridade identificadas. O ISA_Rural_proposto pode ser aplicado na sua totalidade ou ainda na avaliação individual de cada indicador de sua composição. Os resultados da sua aplicação possibilitaram identificar as comunidades com piores condições de salubridade ambiental e os indicadores que requerem maior atenção prioritária nas comunidades estudadas.

## INTRODUÇÃO

A saúde é resultado das condições de vida de uma população, expressando a organização social e econômica do país, tendo como determinantes e condicionantes: a alimentação; a moradia; o saneamento básico; o meio ambiente; o trabalho; a renda; a educação; a atividade física; o transporte; o lazer e o acesso aos bens e serviços essenciais, entre outros^1^.

Assim, essas necessidades básicas individuais e coletivas promovem a salubridade ambiental de uma população. No âmbito internacional, não existe um conceito direto de salubridade ambiental, uma vez que a terminologia é apresentada pela expressão *environmental health* , que corresponde à saúde ambiental e, no Brasil, difere do conceito de salubridade ambiental. No geral, os trabalhos utilizam os termos *health, hygiene* e *cleaning* para abordar a questão salubre. No Brasil, a salubridade ambiental foi definida inicialmente pela Lei estadual nº 7.750, de 31 de março de 1992, no Art. 2º, Inciso II, “como a qualidade ambiental capaz de prevenir a ocorrência de doenças veiculadas pelo meio ambiente e de promover o aperfeiçoamento das condições mesológicas favoráveis à saúde da população urbana e rural”^2^. Esse conceito vem passando por mudanças, conforme apresentado em diversas publicações^3^.

O estudo sobre a salubridade ambiental de um local é importante para mensurar a situação de saúde que determinada população goza em decorrência de suas condições de vida. Portanto, um ambiente salubre pode ser mensurado por meio da determinação da situação de saúde de uma população, influenciada pelas condições socioeconômicas, pela educação, pelo saneamento básico e pelos ambientes em que circula cotidianamente.

Nesse contexto, para determinação da salubridade ambiental, foi proposto o indicador de salubridade ambiental (ISA) pelo Conselho Estadual de Saneamento (Conesan)^6^, a partir do qual tem se adaptado sua composição original, com a inclusão e a exclusão de indicadores e/ou subindicadores e a alteração de seus pesos. Muitas vezes isso ocorre de forma arbitrária ou por meio da replicação de estudos existentes, considerando-se, ou não, as peculiaridades da região analisada^7^. A seleção de indicadores para compor o ISA deve ser feita de forma criteriosa e interrelacionada a seu problema e objetivo de análise. Poucos estudos utilizaram a revisão bibliográfica^10^e empregaram o método Delphi^11 , 12^para a proposição de um índice.

Apesar da boa aceitabilidade do ISA, são poucas as pesquisas existentes sobre a salubridade ambiental em áreas rurais. De um total de 76 estudos sobre o ISA^9^, apenas sete foram aplicados a áreas rurais, onde somente um estudo adaptou o ISA, considerando as relações conceituais de saneamento e saúde. Porém, o objeto de estudo, naquele caso, foram os domicílios rurais, e não o aglomerado rural^13^.

Desse modo, o objetivo deste trabalho foi propor um índice para determinar a salubridade ambiental em aglomerados rurais (ISA_Rural_) e aplicá-lo em comunidades rurais do estado de Goiás.

## MÉTODOS

A metodologia utilizada nesta pesquisa foi realizada em três fases, antecedidas por uma revisão bibliográfica que utilizou as seguintes bases de dados: *Scientific Eletronic Library Online* (SciELO); Periódicos Capes; *Web of Science* e outras ferramentas on-line de busca. Para isso, foram usadas as palavras-chave em inglês e português: *“indicator”* ; *“index”* ; *“salubrity”* ; *“environmental health”* ; *“environmental”* ; *“health”* ; “indicador”; “índice”; “salubridade”; “salubridade ambiental”; “saúde ambiental”; “indicador de salubridade” e “ISA”. O material encontrado forneceu subsídios para a elaboração dos formulários utilizados na primeira e segunda fases.

### Primeira Fase: Análise Prévia para a Proposição de um ISARural

Esta fase foi realizada com o intuito de definir a metodologia a ser aplicada para a proposição de um ISA_Rural_. Para isto, selecionaram-se especialistas em função da sua área de atuação, relacionada ao ISA e a indicadores ambientais ou à saúde ambiental, além da disponibilidade para contribuir com o projeto. Sendo assim, foram escolhidos sete especialistas que pudessem participar das atividades e estar presentes em uma atividade presencial. Com o intuito de nortear e trazer subsídios para as discussões, foi elaborado e aplicado um formulário de entrevista semiestruturado, contendo: programação, finalidade do ISA, conceitos de salubridade ambiental, Manual Básico do ISA^6^e seis perguntas norteadoras (em arquivo complementar)^a^. Após o planilhamento das respostas, houve um encontro presencial, em Goiânia, em 20 de março de 2019, quando foi discutido o tema, culminando com a indicação de um método para ser aplicado na proposição de um ISA_Rural_, além dos indicadores iniciais a serem utilizados para sua composição e a definição de consulta aos especialistas por área de domínio.

### Segunda Fase: Proposição do ISARural

A proposição do ISA foi construída utilizando-se o método Delphi, que pode ser usado para estruturar o processo de comunicação de um grupo de tal forma que ele possa, de forma integrada, lidar com problemas complexos^14^. A proposição foi construída na seguinte sequência:

### Seleção de especialistas

- Grupo 1: composto por 168 especialistas de todas as Unidades Federativas (UF) do Brasil, do Distrito Federal e representantes das comunidades rurais, com suas áreas de atuação relacionadas à pesquisa, que orientaram na escolha e na avaliação dos indicadores do ISA_Rural_.
- Grupo 2: composto por 66 membros formados a partir dos integrantes do Grupo 1 que aceitaram participar da pesquisa e a mais dois pesquisadores da área de saúde ambiental, que, depois, foram subdivididos por áreas de atuação (abastecimento de água; esgotamento sanitário; resíduos sólidos; águas pluviais; saúde ambiental; gestão; e comunidade) e utilizados para a escolha e avaliação dos subindicadores.

- a) 1ª etapa: Seleção dos indicadores
  - 1ª rodada: escolha dos indicadores pré-selecionados pelos especialistas na discussão presencial, bem como sugestão de novos indicadores e subindicadores para cada indicador proposto. Por meio de um formulário com as etapas de contextualização, foi realizada a escolha dos indicadores e a sugestão de subindicadores.
  - 2ª rodada: reavaliação das respostas frente ao que foi respondido pelos outros especialistas e inclusão, ou exclusão, dos indicadores sugeridos na 1ª rodada. Por meio de um formulário contendo os resultados da 1ª rodada, novos indicadores foram sugeridos e avaliados.
- b) 2ª etapa: Avaliação dos indicadores
  - Formulação do ISA_Rural_: apresentação dos indicadores escolhidos e ponderação dos indicadores do ISA_Rural_por meio da aplicação de um formulário contendo os resultados da 1ª e 2ª rodadas.
- c) 3ª etapa: Seleção e avaliação dos subindicadores
  - 1ª rodada: destinada à escolha e ponderação dos subindicadores e sugestão de novos. Contou com a aplicação de formulários com os resultados da primeira rodada de escolha dos indicadores enviados para cada subgrupo de especialistas referente à sua área de atuação, meio pelo qual se fez a apresentação e a análise dos subindicadores sugeridos. Depois, foram selecionados e avaliados os subindicadores de cada ISA_Rural_.
  - 2ª rodada: destinada à reavaliação das respostas dos outros especialistas. Por meio da aplicação de um formulário contendo os resultados da primeira rodada de análise, foram selecionados e avaliados os subindicadores de cada indicador do ISA_Rural_^b^.

O projeto com consulta aos especialistas foi aprovado pelo Comitê de Ética em Pesquisa da Universidade Federal de Goiás (UFG) sob o protocolo nº 3.893.454/2020.

### Terceira Fase: Aplicação do ISARural

A terceira e última fase consistiu na aplicação do ISA_Rural_e na medição e análise da salubridade ambiental em 43 comunidades rurais e tradicionais do estado de Goiás, sendo 16 assentamentos, 21 quilombolas e seis ribeirinhas ( Tabela 3 ). Os dados para o cálculo do ISA foram oriundos do projeto saneamento e saúde ambiental em comunidades rurais e tradicionais de Goiás (SanRural), desenvolvido pela UFG e financiado pela Fundação Nacional de Saúde (Funasa), do qual os autores fazem parte. Os dados foram coletados *in loco* , englobando análise da água, exames de sangue e fezes, aplicação de formulários e checklists para o levantamento das condições de saneamento, saúde, habitação, higiene, uso, ocupação do solo, infraestrutura coletiva e, ainda, das condições socioeconômicas. O projeto foi aprovado pelo Comitê de Ética em Pesquisa da UF Goiás, sob o protocolo nº 2.886.174/2018.

**Tabela 3:** Posição decrescente e valores dos indicadores do ISARural de comunidades rurais do estado de Goiás classificados quanto à sua salubridade.

Nome da comunidade e tipologia	I_AB_	I_ES_	I_MRS_	I_MAP_	I_Saúde_	I_SE_	I_Serviços_	I_CM_	ISA_Rural_
Julião Ribeiro^a^	64,55	65,92	32,59	75,27	82,99	36,50	55,70	85,69	**62,71**
Povoado Veríssimo^c^	77,23	5,26	65,18	48,30	74,88	50,37	98,48	80,51	**58,41**
Tarumã^a^	70,22	55,93	22,09	59,93	70,02	29,73	71,36	80,85	**57,81**
Monte Moriá^a^	60,67	48,09	19,70	52,97	71,47	37,70	56,66	75,68	**52,72**
Itajá II^a^	57,45	25,12	32,70	58,60	79,48	34,60	71,09	82,89	**51,97**
João de Deus^a^	75,10	0,00	33,07	60,07	78,97	27,13	84,72	83,54	**51,49**
Vazante^b^	42,12	18,91	64,24	61,01	65,28	36,92	84,72	82,63	**50,87**
Mesquita^b^	55,18	7,38	38,54	64,04	75,36	46,92	71,60	76,43	**49,20**
Extrema^b^	70,01	2,65	34,91	44,34	71,23	30,25	83,89	79,86	**48,77**
Povoado Vermelho^b^	64,88	19,52	18,72	62,36	69,14	22,96	80,83	73,92	**48,52**
Engenho da Pontinha^a^	64,88	0,00	31,85	55,56	74,97	19,03	84,72	91,42	**48,16**
Lageado^a^	64,88	4,23	24,16	48,51	67,98	23,96	100,00	79,97	**46,89**
Castelo, Retiro e Três Rios^b^	53,69	3,63	23,41	60,28	79,37	28,38	97,62	80,03	**46,71**
Fio Velasco^c^	46,84	37,64	46,24	28,72	64,30	14,34	56,66	74,44	**46,08**
Registro do Araguaia^c^	49,49	6,57	42,09	57,32	65,25	26,13	84,72	82,87	**46,03**
Fortaleza^a^	64,88	0,00	24,86	53,96	70,20	21,50	84,72	82,36	**45,90**
Santa Fé da Laguna^a^	56,46	1,81	24,70	69,48	68,08	31,48	83,89	79,46	**45,81**
Arraial das Pontes^c^	49,71	0,00	56,51	78,21	59,77	18,89	56,66	88,08	**45,71**
Forte^b^	64,88	0,00	17,57	57,72	70,50	32,70	84,72	70,97	**45,21**
Fazenda Santo Antônio da Laguna^b^	53,50	0,00	22,19	58,56	80,06	16,57	83,52	88,87	**44,64**
Queixo Dantas^b^	61,88	0,00	19,70	60,28	72,53	13,45	84,72	80,79	**44,48**
Landi^c^	69,28	0,00	19,70	48,66	70,08	23,65	56,66	84,80	**44,42**
Povoado Moinho^b^	40,61	6,80	33,56	60,08	75,13	28,44	84,72	78,78	**44,21**
Sumidouro^b^	51,93	2,44	30,84	57,55	75,37	26,74	56,66	84,75	**44,03**
Rochedo^a^	37,26	0,00	29,98	57,87	77,70	34,77	100,00	80,19	**43,44**
Céu Azul^a^	43,43	0,00	22,88	52,65	73,95	43,32	100,00	76,32	**43,43**
São Lourenço^a^	44,71	0,00	27,66	57,48	78,33	21,05	99,24	75,32	**43,20**
Piracanjuba^a^	42,12	0,00	37,52	47,25	76,01	31,73	84,72	79,33	**43,19**
Almeidas^b^	58,01	0,00	31,05	51,99	64,06	18,02	84,72	71,90	**42,96**
São Sebastião da Garganta^a^	42,04	0,00	29,62	64,73	66,55	33,46	84,72	80,81	**42,60**
Madre Cristina^a^	51,30	3,97	28,05	50,46	68,45	27,76	74,17	65,89	**41,87**
Olhos d’água^c^	49,14	0,00	19,70	50,74	79,33	4,92	84,72	84,33	**41,26**
Água Limpa^b^	51,30	0,00	24,35	51,87	75,44	23,11	55,76	76,12	**40,96**
Rafael Machado^b^	35,64	0,00	30,84	54,94	70,21	36,60	83,18	77,27	**40,84**
Taquarussu^b^	37,68	0,00	19,70	54,06	77,48	31,75	81,09	60,42	**38,59**
São Domingos^b^	49,99	3,53	19,15	60,26	76,53	18,08	64,13	39,13	**38,16**
Canabrava^b^	24,69	0,00	19,70	55,39	80,48	25,45	83,21	78,95	**37,39**
Quilombo do Magalhães^b^	36,10	0,00	16,89	59,75	73,29	6,27	53,80	84,33	**36,02**
José de Coleto^b^	32,76	0,00	17,51	65,81	63,96	24,48	83,12	42,40	**34,24**
Arraial da Antas^a^	34,80	0,00	24,16	52,76	69,20	6,27	100,00	37,27	**34,24**
Baco Pari^b^	7,02	2,44	20,09	50,01	72,92	14,18	84,72	59,69	**29,86**
Porto Leucádio^b^	8,42	0,00	19,70	56,96	74,59	20,52	56,66	67,14	**29,86**
Pelotas^b^	5,52	0,00	19,70	63,58	83,84	14,68	56,66	51,69	**28,96**
**Média**	49,35	7,49	28,78	56,75	72,90	25,92	78,46	75,30	**44,23**

^a^Assentamento.

^b^Quilombola.

^c^Ribeirinha.

Nota: condições de salubridade ambiental: azul = salubre (de 76 a 100 pontos), verde = média salubridade (de 51 a 75 pontos), laranja = baixa salubridade (de 26 a 50 pontos) e vermelho = insalubre (0 a 25 pontos).

Todos os indicadores e subindicadores que compuseram o ISA_Rural_foram calculados com a utilização do software Microsoft Excel. Os resultados foram apresentados para cada comunidade estudada, bem como da pior para a melhor condição de salubridade ambiental entre elas, conforme as seguintes faixas de pontuações: insalubre (entre 0 e 25), de baixa salubridade (de 26 a 50), de média salubridade (de 51 a 75) ou salubre (de 76 a 100)^3^.

## RESULTADOS E DISCUSSÃO

### Análise Prévia para a Proposição de um ISARural

O encontro presencial se iniciou com a discussão sobre as respostas de 57,14% dos especialistas consultados na primeira fase. A partir dos conceitos existentes de salubridade ambiental e tendo como principal referência o conceito utilizado atualmente pela Funasa^5^, foi discutido e proposto, junto aos especialistas que contribuíram no estudo, que “salubridade ambiental consiste na situação de saúde que determinada população goza em decorrência das condições socioeconômicas e ambientais em que vive”, utilizado como referência para a determinação dos indicadores e dos subindicadores e suas ponderações.

Em função da diversidade do meio rural, definiu-se que a proposição do ISA_Rural_seja para aglomerados rurais, e não para o meio rural como um todo. O Instituto Brasileiro de Geografia e Estatística (IBGE) define aglomerado rural como unidades domiciliares com edificações adjacentes, isto é, 50 metros ou menos de distância entre si e com características de permanência^15^. Neste sentido, o ISA_Rural_poderá ser aplicado nos setores censitários:1b, 2 e 4 (aglomerações próximas do urbano); 3 (aglomerações mais adensadas isoladas), 5, 6 e 7 (aglomerações menos adensadas isoladas), definidos no programa nacional de saneamento rural (PNSR)^16^, um dos três programas do plano nacional de saneamento básico (Plansab)^17^.

Por meio do consenso dos especialistas, o método escolhido como o mais adequado foi o Delphi, que foi desenvolvido em três etapas: 1) escolha e/ou complementação dos indicadores sugeridos na discussão presencial; 2) avaliação dos indicadores, e 3) escolha e avaliação dos subindicadores. Para o início da consulta aos especialistas foram sugeridos sete indicadores: indicador de abastecimento de água (I_AB_); indicador de esgotamento sanitário (I_ES_); indicador de resíduos sólidos (I_RS_); indicador de drenagem (I_DR_); indicador de saúde (I_Saúde_); indicador socioeconômico (I_SE_) e indicador de serviços (I_Serviços_). Por fim, definiu-se que os especialistas deveriam ser selecionados e divididos por áreas de atuação, compondo sete grupos, sendo quatro relacionados ao saneamento básico, um à saúde ambiental e outros dois à gestão ambiental e às comunidades rurais. Os dois últimos grupos têm a função de permitir a análise da composição dos indicadores e de revelar, pelos representantes das comunidades, as particularidades e limitações das áreas rurais. Assim, a fase de análise prévia cumpriu com a tarefa de definir a metodologia a ser utilizada na proposição do ISA_Rural_.

### Proposição do ISARural

Após o consenso obtido na fase anterior, iniciou-se a proposição do ISA_Rural_empregando o método Delphi, que foi dividido em três etapas conforme descritas na Tabela 1 . Nela é apresenta o número de especialistas convidados, a frequência e o tempo de retorno, bem como as UF e o Distrito Federal sem retorno, representando, no final da composição do ISA_Rural_, 70,4% de participação, que, em função das dimensões geográficas do país, foi considerada como excelente.

**Tabela 1:** Etapas da aplicação do método Delphi, com o número de especialistas convidados, frequência de retorno, período e unidades federativas brasileiras sem retorno dos especialistas.

Etapas do método Delphi		Quantidade de especialistas convidados	Retorno dos especialistas (%)	Período (dias)	Participação de representantes das UF (%)	UF sem retorno de resposta
1ª	1ª rodada para escolha e sugestão de indicadores e sugestão de subindicadores	168	38,1	52	85,2	MT, MS, PA e PE
	2ª rodada para escolha dos indicadores	64	84,4	46	77,8	MT, MS, PA, PE, AM e RS
2ª	Ponderação dos pesos dos indicadores	54	87,0	37	70,4	MT, MS, PA, PE, AM, RS, ES e SE
3ª	1ª rodada para análise, escolha e ponderação dos subindicadores	66	60,6	60	74,1	MT, MS, PA, PE, AM, AL e ES
	2ª rodada para análise e ponderação dos subindicadores	40	87,5	40	70,4	MT, MS, PA, PE, AM, AL, ES e RS

UF: Unidade Federativa Brasileira; AL: Alagoas; AM: Amazonas; ES: Espírito Santo; MT: Mato Grosso; MS: Mato Grosso do Sul; PA: Pará; PE: Pernambuco; RS: Rio Grande do Sul; SE: Sergipe.

A frequência de concordância dos sete indicadores, definidos na análise prévia e sugeridos na 1ª e 2ª rodadas de seleção dos indicadores do ISA_Rural_( Tabela 1 ), está apresentada na Figura 1 , junto à frequência de sugestão de três novos indicadores sugeridos na 1ª rodada e a porcentagem de concordância de inclusão deles. Na 1ª rodada, os indicadores I_AB_, I_ES,_I_RS_e I_Saúde_obtiveram frequência de concordância de 100%, tendo apenas algumas ressalvas de concordâncias parciais, tal como a inclusão da palavra “manejo” no I_RS_, passando para indicador de manejo de resíduos sólidos (I_MRS_). A maioria dos especialistas concordou, total ou parcialmente, com o I_DR_(89,06%), I_SE_(98,44%) e I_Serviços_(79,69%).

**Figura 1:** Frequência de concordância da permanência e da inclusão dos indicadores na composição do ISARural obtida na primeira e segunda rodadas da primeira etapa da aplicação do método Delphi.

I_AB_: indicador de abastecimento de água; I_ES_: indicador de esgotamento sanitário; I_RS_: indicador de resíduos sólidos; I_MRS:_indicador de manejo de resíduos sólidos; I_DR_: indicador de drenagem; I_MAP_: indicador de manejo de águas pluviais; I_Saúde_: indicador de saúde; I_SE_: indicador socioeconômico; I_Serviços_: indicador de serviços; I_CM_: indicador de condições de moradia; I_EL_: indicador de energia elétrica; I_CV_: indicador de controle de vetores; 1ª R: primeira rodada; 2ª R: segunda rodada.

Em relação ao I_DR_, os especialistas que não concordaram com sua inclusão (10,94%) justificaram que sua relevância é apenas para áreas urbanas, pois, para a área rural, a drenagem é um processo natural, e que a Lei Federal nº 11.455/2007^18^contempla apenas as áreas urbanas. Todavia, o manejo das águas pluviais foi considerado nos indicadores do PNSR^16^. Para esse indicador, também foi sugerido a inclusão da palavra “manejo”, tendo como referência os componentes do saneamento rural do PNSR, passando para indicador de manejo de águas pluviais (I_MAP_) no lugar do I_DR_. Quanto ao I_SE_, apenas um especialista não concordou com a sua inclusão, não apresentando, porém, justificativas. Já para o I_Serviços_, houve justificativas de não concordância por ele ser um indicador muito abrangente, apresentar dificuldade para a obtenção dos dados e por estar contemplado nos indicadores anteriores. Nesta rodada, foi observada a sugestão de mais 21 indicadores diferentes dos sete iniciais, sendo que foi considerado relevante aquele indicador sugerido por dois ou mais especialistas, resultando em três indicadores: 1) indicador de condições de moradia (I_CM_); 2) indicador de energia elétrica (I_EL_); e 3) indicador de controle de vetores (I_C__V_) ( Figura 1 ).

Ainda na 1ª rodada, os subindicadores sugeridos pelos especialistas foram separados em grupos que englobassem a mesma temática. Os que obtiveram maior frequência foram utilizados na proposição dos subindicadores na 3ª etapa da aplicação do método Delphi.

Na 2ª rodada, todos os questionamentos e observações foram apresentados aos especialistas, com a opção de realizarem uma nova análise sobre a composição do ISA_Rural_. Dentre os dez indicadores sugeridos, oito apresentaram frequência superior a 70% de concordância (total e parcial), os quais foram mantidos e levados para a ponderação da fórmula. Desse modo, considerou-se que os indicadores I_EL_e I_CV_já estavam contemplados pelos demais, sendo, assim, retirados do índice. O ISA_Rural_ficou definido pelo uso de oito indicadores, quatro relacionados aos componentes do saneamento básico, um à saúde, um às condições socioeconômicas, um aos serviços ofertados nos aglomerados rurais e um às condições de moradia. Desta forma, permaneceram como indicadores: I_AB_; I_ES_; I_MRS_; I_MAP_; I_Saúde_; I_SE_; I_Serviços;_e I_CM_. Os indicadores de saneamento básico e saúde somaram 75,81% dos pesos do ISA_Rural_.

Na rodada seguinte, relativa à 2ª etapa da aplicação do método Delphi, foi realizada a ponderação dos pesos pelos especialistas para cada um os indicadores, resultando nos seguintes valores médios e desvios padrão para cada indicador: I_AB_= 22,82 ± 7,45; I_ES_= 19,44 ± 5,29; I_MRS_= 13,16 ± 4,01; I_MAP_= 7,82 ± 3,39; I_Saúde_= 12,55 ± 4,85; I_SE_= 8,70 ± 3,92; I_Serviços_= 6,35 ± 2,94 e I_CM_= 9,16 ± 4,62. O ISA_Rural_resultou do valor médio dos pesos atribuídos pelos especialistas para cada indicador, originando a Equação 1,


$${ISA}_{\text{Rural }}=0,2282I_{AB}+0,1944I_{ES}+0,1316I_{MRS}+0,0782I_{MAP}+0,1255I_{\text{Saúde }}+0,0870I_{SE}+0,0635I_{\text{Serviços }}+0,0916I_{CM}$$ (1)

Legenda: indicador de abastecimento de água = I_AB_; indicador de esgotamento sanitário = I_ES_; indicador de manejo de resíduos sólidos = I_MRS_; indicador de manejo de águas pluviais = I_MAP_; indicador de saúde = I_SAÚDE_; indicador socioeconômico = I_SE_; indicador de serviços = I_Serviços_; e indicador de condições de moradia = I_CM_.

Para a 1ª rodada da 3ª etapa da aplicação do método Delphi, e a partir dos grupos de subindicadores que obtiveram maior percentagem de sugestão na 1ª rodada da 1ª etapa, consultou-se a bibliografia técnico-científica específica, levando-se em consideração o conceito de salubridade ambiental, meio pelo qual se formulou uma relação de subindicadores enviados para consulta aos especialistas. A frequência de concordância da inclusão de subindicadores das fórmulas e das pontuações está apresentada na Figura 2 .

**Figura 2:** Frequência de concordância da inclusão dos subindicadores de cada indicador, bem como das fórmulas e pontuações sugeridas na primeira rodada.

I_AB_: indicador de abastecimento de água; I_ES_: indicador de esgotamento sanitário; I_MRS_: indicador de manejo de resíduos sólidos; I_MAP_: indicador de manejo de águas pluviais; I_Saúde_: indicador de saúde; I_SE_: indicador socioeconômico; I_Serviços_: indicador de serviços; I_CM_: indicador de condições de moradia; I_AA_: subindicador de abastecimento adequado de água no domicílio; I_QA_: subindicador de qualidade da água; I_FA_: subindicador de frequência no abastecimento de água; I_DE_: subindicador de destinação adequada de excretas; I_DAS_: subindicador de destinação adequada de águas servidas; I_CRS_: subindicador de coleta adequada de resíduos sólidos; I_DRS_: subindicador de destinação adequada de resíduos sólidos; I_SRS_: subindicador de separação dos resíduos sólidos; I_DEA_: subindicador de destinação adequada de embalagens de agrotóxicos; I_APV_: subindicador de manejo de águas pluviais adequados nas vias; I_UV_: subindicador de dificuldade ou impossibilidade de utilização das vias de acesso; I_CES_: subindicador de controle de escoamento superficial; I_IA_: subindicador de ocorrência de inundação e alagamento; I_E_: subindicador de erosões; I_US_: subindicador de uso do solo; I_DD_: subindicador de ocorrência de diarreia; I_DH_: subindicador de ocorrência de hepatite A; I_VD_: subindicador de dengue; I_VE_: subindicador de esquistossomose; I_VL_: subindicador de leptospirose; I_MI_: subindicador de mortalidade infantil; I_PTA_: subindicador de tratamento da água domiciliar; I_PHM_: subindicador de higienização das mãos; I_RF_: subindicador de renda *per capita* familiar; I_ECF_: subindicador de escolaridade do chefe de família; I_ED_: subindicador de educação; I_E_: subindicador de educação; I_S_: subindicador de saúde; I_EE_: subindicador de energia elétrica; I_MC_: subindicador de meio de comunicação; I_TP_: subindicador de transporte público; I_MP_: subindicador de material usado na parede; I_PA_: subindicador de piso adequada; I_CA_: subindicador de cobertura adequada; I_B_: subindicador de banheiro; I_RA_: subindicador de reservação interna adequada de água.

Os subindicadores do I_AB_, I_MRS_, I_SE_e I_CM_obtiveram frequência de concordância de inclusão de 100%, com algumas ressalvas de adequações nas descrições das fórmulas e ponderações.

Nos subindicadores do I_ES_, apenas dois especialistas (7,1%) não concordaram com a inclusão, justificando que não seria necessário separar o esgoto sanitário em excretas e águas servidas. Porém, nos estudos aplicados em áreas rurais^13^, essa separação foi considerada relevante. Logo, esses subindicadores foram mantidos para a próxima rodada, apenas com pequenas alterações nas fórmulas e pontuações, de acordo com as sugestões.

A respeito do I_MAP_, metade dos subindicadores (I_APV,_I_IA_e I_E_) obtiveram concordância em 100%, e a outra metade obteve frequência de concordância de 87,5% para I_UV_, 91,7% para I_CES_e 95,8% para I_US_. A justificativa foi a irrelevância desses indicadores, influenciando também as respostas obtidas nas fórmulas e pontuações.

Quanto ao I_Serviços_, somente os subindicadores I_EE_e I_TP_não obtiveram concordância de inclusão em 100%, ficando em 88,9%. Nas descrições das fórmulas, a discordância (11,1%) ocorreu nos subindicadores I_E_, I_S_, I_EE_e I_TP_, entre os quais foi sugerida a inclusão do critério de atendimento dos serviços nos subindicadores I_E_e I_S_.

Os subindicadores do I_Saúde_, apesar de terem obtido concordância de inclusão superior a 80%, apresentaram diversas considerações nas descrições das fórmulas. Uma delas foi a modificação da forma de amostragem, de domicílio para habitantes, obtendo a ocorrência da doença. Por ela modificar toda a forma de cálculo, as alterações sugeridas para avaliação na 2ª rodada da 3ª etapa foram apresentadas aos especialistas. Para os demais indicadores, mostrou-se somente a opção de ponderação dos subindicadores.

Na última rodada, foram ponderados todos os subindicadores com a média dos pesos atribuídos e também se obteve a frequência de concordância das alterações nas fórmulas dos subindicadores do I_Saúde_. Apenas um especialista discordou do subindicador de tratamento da água domiciliar (I_PTA_); os demais concordaram integralmente. As fórmulas finais dos indicadores e seus respectivos subindicadores e pontuação estão apresentadas na Tabela 2 .

**Tabela 2:** Fórmulas para o cálculo dos indicadores IAB, IES, IMRS, IMAP, ISaúde, ISE, IServiços e ICM, que compõem o ISARural e seus subindicadores, com descrição e pontuação.

$I_{AB}=0,4212I_{AA}+0,3512I_{QA}+0,2277I_{FA}$			
Subindicador	Fórmula	Pontuação	Descrição
Abastecimento adequado de água no domicílio (I_AA_)	$I_{AA}=\frac{Draa}{Drt}\times 100\%$	Pela fórmula	D_raa_= número de domicílios do aglomerado rural abastecidos por rede de distribuição de água, com canalização interna no domicílio ou na propriedade, ou por poço, nascente ou cisterna de captação de água de chuva, com canalização interna D_rt_= número de domicílios totais do aglomerado rural
Qualidade da água (I_QA_)	$I_{QA}=\frac{NAA}{NRA}\times 100\%$	$\begin{array}{c} I_{\text{oA }}=100\%-\text{ Pontuação }=100\\ I_{\text{oA }}=95\text{ a }100\%\text{ - Pontuação }=80\\ I_{\text{oA }}=85\text{ a }95\%\text{ - Pontuação }=60\\ I_{\text{QA }}=70\text{ a }85\%\text{ - Pontuação }=40\\ I_{\text{oA }}=50\text{ a }70\%\text{ - Pontuação }=20\\ I_{\text{oA }}<50\%\text{ - Pontuação }=0 \end{array}$	N_AA_= quantidade de amostras conformes a valores de qualidade aceitáveis da água relativos à colimetria, ao cloro e à turbidez N_RA_= quantidade de amostras realizadas
Frequência no abastecimento de água (I_FA_)	$I_{FA}=\frac{\mathrm{Drfa}}{Drt}\times 100\%$	Pela fórmula	D_rfa_= número de domicílios rurais em que nunca ou raramente (1 vez por mês) falta água D_rt_= número de domicílios totais do aglomerado rural
$I_{ES}=0,6349I_{DE}+0,3651I_{DAS}$			
**Subindicador**	**Fórmula**	**Pontuação**	**Descrição**
Destinação adequada de excretas (I_DE_)	$I_{DE}=\frac{\text{ Dre }}{\text{ Drt }}\times 100\%$	Pela fórmula	D_re_= número de domicílios do aglomerado rural atendidos por rede coletora seguida de tratamento, fossa séptica ou tecnologias de tratamento de esgoto na zona rural para excretas D_rt_= número de domicílios totais do aglomerado rural
Destinação adequada de águas servidas (I_DAS_)	$I_{DAS}=\frac{\text{ Dras }}{\text{ Drt }}\times 100\%$	Pela fórmula	D_ras_= número de domicílios do aglomerado rural atendidos por rede coletora seguida de tratamento, fossa séptica ou tecnologias de tratamento de esgoto na zona rural para águas servidas D_rt_= número de domicílios totais do aglomerado rural
$I_{\text{MRS }}=0,2817I_{\text{CRS }}+0,2985I_{\text{DRS }}+0,1970I_{\text{SRS }}+0,2228I_{DEA}$			
**Subindicador**	**Fórmula**	**Pontuação**	**Descrição**
Coleta adequada de resíduos sólidos (I_CRS_)	$I_{CRS}=\frac{Drc}{Drt}\times 100\%$	Pela fórmula	D_rc_= número de domicílios do aglomerado rural atendido por sistemas de coleta direta ou indireta de resíduos sólidos com frequência de no mínimo uma vez por semana D_rt_= número de domicílios totais do aglomerado rural
Destinação adequada de resíduos sólidos (I_DRS_)	$I_{DRS}=\left(1-\frac{Drd}{Drt}\right)\times 100\%$	Pela fórmula	D_rd_= número de domicílios do aglomerado rural que enterram, queimam ou destinam a céu aberto os resíduos sólidos D_rt_= número de domicílios totais do aglomerado rural
Separação dos resíduos sólidos (I_SRS_)	$I_{SRS}=\frac{DrS}{Drt}\times 100\%$	Pela fórmula	D_rs_= número de domicílios do aglomerado rural que realizam a separação dos resíduos sólidos D_rt_= número de domicílios totais do aglomerado rural
Destinação adequada de embalagens de agrotóxicos (I_DEA_)	$I_{DEA}=\frac{\text{ Drea }}{\text{ Drta }}\times 100\%$	Pela fórmula	D_rea_= número de domicílios do aglomerado rural que devolvem as embalagens de agrotóxicos ao fabricante, vendedor do produto ou a um posto de entrega D_rta_= número de domicílios totais do aglomerado rural que utilizam agrotóxicos
$I_{MAP}=0,1639I_{APV}+0,1308I_{UV}+0,1580I_{CES}+0,2133I_{IA}+0,1721I_{E}+0,1619I_{US}$			
**Subindicador**	**Fórmula**	**Pontuação**	**Descrição**
Manejo de águas pluviais adequados nas vias (I_APV_)	$I_{APV}=\frac{Drvp}{Drt}\times 100\%$	Pela fórmula	D_rvp_= número de domicílios do aglomerado rural localizados em vias com pavimentação, meio fio e bocas de lobo D_rt_= número de domicílios totais do aglomerado rural
Dificuldade ou impossibilidade de utilização das vias de acesso (I_UV_)	$I_{UV}=\frac{\mathrm{Drac}}{Drt}\times 100\%$	Pela fórmula	D_rac_= número de domicílios do aglomerado rural que não apresentaram dificuldade de acesso às suas casas nos últimos cincos anos D_rt_= número de domicílios totais do aglomerado rural
Controle de escoamento superficial (I_CES_)	$I_{CES}=\frac{\text{ Drce }}{Drt}\times 100\%$	Pela fórmula	D_rce_= número de domicílios do aglomerado rural com dispositivos de controle de escoamento superficial excedente, como curva de nível, canaleta ou valeta, ou outros D_rt_= número de domicílios totais do aglomerado rural
Ocorrência de inundação e alagamento (I_IA_)	$I_{IA}=\frac{\text{ Dria }}{\text{ Drt }}\times 100\%$	Pela fórmula	D_ria_= número de domicílios do aglomerado rural sem ocorrência de inundações, nos últimos cinco anos, e alagamento D_rt_= número de domicílios totais do aglomerado rural
Erosões (I_E_)	$I_{E}=\frac{\text{ Dre }}{Drt}\times 100\%$	Pela fórmula	D_re_= número de propriedades do aglomerado rural que não apresentaram erosões D_rt_= número de propriedades totais do aglomerado rural
Uso do solo (I_US_)	$I_{US}=\text{ Cus }\times 100\%$	Pela fórmula	Uso do solo predominante do aglomerado rural (C_us_), critério: vegetação nativa: 1; pastagem: 0,5; agricultura: 0,25; solo exposto: 0
$I_{\text{Saúde }}=0,1557I_{DD}+0,1292I_{DH}+0,1038I_{VD}+0,1018I_{VE}+0,0941I_{VL}+0,1710I_{MI}+0,1414I_{PTA}+0,1030I_{PHM}$			
**Subindicador**	**Fórmula**	**Pontuação**	**Descrição**
Ocorrência de diarreia (I_DD_)	$I_{DD}=\left(1-\frac{Hrdd}{Hrt}\right)\times 100\%$	Pela fórmula	Hrdd = número de habitantes residentes no aglomerado rural com diarreia no último mês. H_rt_= número total de habitantes residentes no aglomerado rural
Ocorrência de Hepatite A (I_DH_)	$I_{DH}=\left(1-\frac{Hrdh}{Hrt}\right)\times 100\%$	Pela fórmula	H_rdh_= número habitantes residentes no aglomerado rural diagnosticados com hepatite A H_rt_= número total de habitantes residentes no aglomerado rural
Dengue (I_VD_)	$I_{VD}=\left(1-\frac{Hrvd}{Hrt}\right)\times 100\%$	Pela fórmula	H_rvd_= número de habitantes residentes no aglomerado rural diagnosticados com dengue, zika, chikungunya ou febre amarela H_rt_= número total de habitantes residentes no aglomerado rural
Esquistossomose (I_VE_)	$I_{VE}=\left(1-\frac{\text{ Hrve }}{Hrt}\right)\times 100\%$	Pela fórmula	H_rve_= Número de habitantes residentes no aglomerado rural diagnosticados com esquistossomose H_rt_= Número total de habitantes residentes no aglomerado rural
Leptospirose (I_VL_)	$I_{VL}=\left(1-\frac{Hrvl}{Hrt}\right)\times 100\%$	Pela fórmula	H_rvl_= número de habitantes residentes no aglomerado rural diagnosticados com leptospirose H_rt_= número total de habitantes residentes no aglomerado rural
Mortalidade infantil (I_MI_)	$I_{MI}=\left(1-\frac{Crmi}{Crt}\right)\times 100\%$	Pela fórmula	C_rmi_= número de crianças menores de 1 ano residentes no aglomerado rural com ocorrência de óbito no último ano C_rt_= número total de crianças menores de 1 ano residentes no aglomerado rural
Tratamento da água domiciliar (I_PTA_)	$I_{PTA}=\frac{Drta}{Drt}\times 100\%$	Pela fórmula	D_rta_= número de domicílios do aglomerado rural que realizam algum tratamento na água para beber, como filtração, fervura ou desinfecção D_rt_= número de domicílios totais do aglomerado rural
Higienização das mãos (I_PHM_)	$I_{PHM}=\frac{\frac{Hrmr}{Hrt}+\frac{Hrmb}{Hrt}}{2}\times 100\%$	Pela fórmula	H_rmr_= número de habitantes residentes no aglomerado rural que sempre lavam as mãos com água e sabão antes das refeições H_rmb_= número de habitantes residentes no aglomerado rural que sempre lavam as mãos com água e sabão após o uso do banheiro H_rt_= número total de habitantes residentes no aglomerado rural
$I_{SE}=0,4389I_{RF}+0,2556I_{ECF}+0,3056I_{ED}$			
**Subindicador**	**Fórmula**	**Pontuação**	**Descrição**
Renda *per capita* familiar (I_RF_)	$I_{RF}=\frac{\mathrm{Drrf}}{\mathrm{Drt}}\times 100\%$	Pela fórmula	D_rrf_= número de domicílios do aglomerado rural com renda mensal *per capita* familiar maior ou igual a um salário-mínimo D_rt_= número de domicílios totais do aglomerado rural
Escolaridade do chefe de família (I_ECF_)	$I_{ECF}=\frac{\text{ Drecf }}{\text{ Drt }}\times 100\%$	Pela fórmula	D_recf_= número de domicílios do aglomerado rural cujo chefe de família possui pelo menos o ensino fundamental completo D_rt_= número de domicílios totais do aglomerado rural
Educação (I_ED_)	$I_{ED}=\sqrt[{4}]{Epa^{*}Fpj^{2}}$	Pela fórmula	Escolaridade da população adulta (E_pa_) = percentual de habitantes do aglomerado rural com 18 anos ou mais de idade com o ensino fundamental completo Fluxo escolar da população jovem (F_pj_): média aritmética (1) do percentual de crianças de 5 a 6 anos frequentando a escola; (2) do percentual de jovens de 11 a 13 anos frequentando os anos finais do ensino fundamental regular; (3) do percentual de jovens de 15 a 17 anos com ensino fundamental completo, e (4) do percentual de jovens de 18 a 20 anos com ensino médio completo
$I_{\text{Serviços }}=0,2222I_{E}+0,2806I_{S}+0,2000I_{EE}+0,1444I_{MC}+0,1528I_{TP}$			
**Subindicador**	**Fórmula**	**Pontuação**	**Descrição**
Educação (I_E_)	$I_{E}=E\times 100\%$	Pela fórmula	Educação básica no aglomerado rural (E), critério: o aglomerado rural é atendido por serviço de educação básica (escola no aglomerado rural ou disponibilidade de transporte escolar até uma unidade de educação básica) = 1; o aglomerado rural não é atendido serviço de educação pública = 0
Saúde (I_S_)	$I_{S}=S\times 100\%$	Pela fórmula	Saúde no aglomerado rural (S), critério: o aglomerado rural é atendido por serviço de saúde (posto de atendimento ou agentes comunitários) = 1; o aglomerado rural não é atendido por serviço de saúde pública = 0
Energia elétrica (I_EE_)	$I_{EE}=\frac{\text{ Dree }}{\text{ Drt }}\times 100\%$	Pela fórmula	D_ree_= número de domicílios do aglomerado rural com energia elétrica. D_rt_= número de domicílios totais do aglomerado rural
Meio de comunicação (I_MC_)	$I_{MC}=\frac{Drmc}{Drt}\times 100\%$	Pela fórmula	D_rmc_= número de domicílios do aglomerado rural com acesso a telefone, rádio, televisão ou internet D_rt_= número de domicílios totais do aglomerado rural
Transporte público (I_TP_)	$I_{TP}=Tp\times 100\%$	Pela fórmula	Transporte público no aglomerado rural (T_p_), critério: o aglomerado rural é atendido por serviço de transporte público = 1; o aglomerado rural não é atendido por serviço de transporte público = 0
$I_{CM}=0,1430Imp+0,1505I_{PA}+0,1555I_{CA}+0,3125I_{B}+0,2385I_{RA}$			
**Subindicador**	**Fórmula**	**Pontuação**	**Descrição**
Material usado na parede (I_MP_)	$I_{MP}=\frac{Drmp}{Drt}\times 100\%$	Pela fórmula	D_rmp_= número de domicílios do aglomerado rural com parede em alvenaria e reboco D_rt_= número de domicílios totais do aglomerado rural
Piso adequada (I_PA_)	$I_{PA}=\frac{Drpa}{Drt}\times 100\%$	Pela fórmula	D_rpa_= número de domicílios do aglomerado rural com piso impermeável ou que facilite a adequada higienização D_rt_= número de domicílios totais do aglomerado rural
Cobertura adequada (I_CA_)	$I_{CA}=\frac{Drca}{Drt}\times 100\%$	Pela fórmula	D_rca_= número de domicílios do aglomerado rural com cobertura em telha ou outro recurso adequado ao isolamento das águas de chuva e ao isolamento térmico D_rt_= número de domicílios totais do aglomerado rural
Banheiro (I_B_)	$I_{B}=\frac{Drb}{Drt}\times 100\%$	Pela fórmula	D_rb_= número de domicílios do aglomerado rural que possuem banheiro com vaso sanitário e chuveiro D_rt_= número de domicílios totais do aglomerado rural
Reservação interna adequada de água (I_RA_)	$I_{RA}=\frac{\text{ Drra }}{Drt}\times 100\%$	Pela fórmula	D_rra_= número de domicílios do aglomerado rural com reservatório interno de água (caixa d’água) tampado e higienizado a cada seis meses D_rt_= número de domicílios do aglomerado rural com reservatório interno

Ao comparar a proposição final do ISA_Rural_com estudos encontrados na bibliografia específica, constatou-se que nenhum contempla, de forma integral, todos os indicadores. A separação dos especialistas por área de atuação trouxe a formulação de subindicadores essenciais e com especificidades, exigindo dados de simples obtenção para o cálculo. Alguns já são exigidos do poder público pelo PNSR^16^, os outros são possíveis de se obter por meio de questionários aplicados, podendo ser empregados pelos agentes comunitários de saúde, os capacitando, assim como sugerido em Bernardes, Bernardes e Gunther^13^.

### Aplicação do ISARural

A aplicação do ISA_Rural_proposto permitiu constatar que apenas 14% das comunidades são de média salubridade, com os assentamentos ocupando cinco das seis primeiras colocações. Nas demais comunidades (86%), há baixa salubridade ( Tabela 3 ), com 61,9% das comunidades quilombolas abaixo da média. A posição decrescente das comunidades rurais de Goiás, segundo os resultados do ISA_Rural_e dos seus indicadores, está apresentada na Tabela 3 .

Analisando-se separadamente o I_AB_, apenas a comunidade Povoado Veríssimo (77,23%) enquadrou-se como salubre, e 48,84% das comunidades foram classificadas com média salubridade. As demais, 39,5%, apresentaram condições de baixa salubridade e 9,3% de insalubridade. No diagnóstico do PNSR^16^, para as aglomerações menos adensadas isoladas, 46,3% dos habitantes possuem atendimento adequado no componente abastecimento de água, estando próximo ao valor médio de 49,35 pontos ( Tabela 3 ) obtido para o I_AB_. A baixa salubridade ocorreu devido principalmente à qualidade da água de abastecimento, com a presença de *E. coli* na maioria das amostras de água analisadas, resultando em desacordo com o Anexo XX da Portaria de Consolidação nº 5 do Ministério da Saúde^19^. A presença de *E. coli* na água consumida pela população em comunidades rurais é relatada em trabalhos científicos nacionais e internacionais^20 , 21^, sendo algo recorrente e que requer atenção do poder público. Na maioria das situações, a desinfecção intradomiciliar da água com solução de hipoclorito de sódio diminuiria consideravelmente a contaminação^22^e, consequentemente, melhoraria a salubridade. Dentre os indicadores que compõem o ISA_Rural_, o I_ES_foi aquele que apresentou os piores resultados, presente em 90,7% das comunidades em situação de insalubridade, o que requer maior atenção do poder público. Essa condição decorre da utilização, na grande maioria dos domicílios, de uma fossa rudimentar como solução para o esgotamento sanitário. Esse resultado corrobora com os dados apresentados no PNSR, de que apenas 15,2% dos habitantes destinam seus efluentes adequadamente^16^, e com o estudo de Roland et al.^23^Estudo realizado em comunidades ribeirinhas da Amazônia concluiu que uma das características que mais contribuem para a situação de insalubridade e baixa salubridade é a precariedade dos domicílios em relação à destinação adequada de excretas e águas cinzas^13^. Somente duas comunidades (4,65%) foram classificadas com média salubridade, e outras duas (4,65%) com baixa salubridade.

Outro componente do saneamento básico preocupante é o manejo dos resíduos sólidos, representado pelo I_MRS_, presente em apenas 6,98% das comunidades atendidas, em mais de 80% dos domicílios por coleta direta ou indireta de resíduos sólidos. Apesar da grande maioria dos domicílios das comunidades separar os resíduos, eles não têm destinos adequados, e a queima é a principal forma de destinação, semelhante à situação apresentada no diagnóstico do PNSR^16^e em outros estudos^24^. Além de ser proibida pelo artigo 47 da Política Nacional de Resíduos Sólidos^25^, essa prática, a depender da composição dos resíduos, pode liberar gases tóxicos, bem como não reduz todos os tipos de resíduos, podendo contribuir para a proliferação de doenças e influenciar a qualidade de vida da população^23^. Diante do exposto sobre o I_MRS_, 53,49% das comunidades se enquadram como insalubres, 39,53% com baixa salubridade e 6,98% com média salubridade.

Em relação ao I_MAP_, apenas a comunidade ribeirinha Arraial da Ponte, representando 2,33% do total de comunidades analisadas, foi classificada como salubre. Essa condição foi caracterizada pela presença de pavimentação nas ruas, com meio fio e bocas de lobo (dispositivo que permite as águas de chuvas serem drenadas para a rede/galeria de águas pluviais), atendendo 50% da comunidade. Quanto às demais, 76,74% foram classificadas com média salubridade, e 20,93% com baixa salubridade. O manejo de águas pluviais é o único componente do saneamento básico em que não foi possível diagnosticar a situação vigente das áreas rurais do Brasil pelo PNSR^16^, pois o IBGE^26^não dispõe de dados suficientes para tal análise, sendo uma das maiores barreiras para a realização de estudos nesse componente do saneamento básico, o que detém o direcionamento adequado de políticas públicas para a resolução de problemas relacionados a infraestrutura^23^.

O I_Saúde_foi o terceiro indicador a apresentar os melhores resultados na pesquisa. A situação salubre foi registrada em 30,2% das comunidades e média salubridade em 69,8%. Isso se deve principalmente ao fato de os habitantes das comunidades não terem sido diagnosticados por um profissional de saúde com esquistossomose e/ou leptospirose, com exceção de um habitante da comunidade Julião Ribeiro, além de não ter ocorrido nenhum óbito de crianças menores de 1 ano nessas comunidades. Entretanto, muitos habitantes das comunidades testaram positivo para a hepatite A, corroborando outro estudo sobre assentamentos rurais no sudoeste de Goiás em que 82,20% dos residentes foram detectados com anticorpos para o vírus^27^, principal fator de diminuição da salubridade nesse indicador.

O I_SE_foi o segundo indicador a apresentar os piores resultados de salubridade. Desse modo, 48,84% das comunidades apresentaram situação de insalubridade e 51,16% de baixa salubridade devido à baixa escolaridade e à renda *per capita* mensal dos habitantes. Isso consolidou os dados apresentados no PNSR^16^e as análises de que quanto mais baixos os níveis de escolaridade e renda, piores são as soluções adotadas em saneamento básico^28^.

De modo geral, o I_Serviços_apresentou os melhores resultados, com 65,12% das comunidades salubres e 34,88% com média salubridade. Essa condição é justificada pelo fato de 100% das comunidades terem sido atendidas por serviço de educação básica, 69,77% por serviço de saúde, e, em mais de 90% dos domicílios, 93% e 62,8% delas terem acesso, respectivamente, à energia elétrica e aos meios de comunicação. O Programa Nacional de Universalização do Acesso e Uso da Energia Elétrica^29^, responsável pela evolução da universalização do acesso à energia, com prazo limite para 2022, já foi prorrogado por diversas vezes. Portanto, ele produz, e certamente produzirá, melhorias em dinâmicas sociais e econômicas para as comunidades que ainda não são integralmente atendidas por esse serviço fundamental^30^.

Por fim, o I_CM_foi o segundo indicador a apresentar os melhores resultados de salubridade, com 67,44% das comunidades em situação salubre, 25,58% com média salubridade e 6,98% com baixa salubridade. Em geral, as comunidades possuem casas com parede, piso e cobertura adequados, incluindo o banheiro. Porém, seus reservatórios de água estão em condições inadequadas, podendo ser um dos fatores que contribui para a baixa qualidade da água e por serem locais de contaminação^31^.

## CONCLUSÕES

O ISA_Rural_proposto está em consonância com o conceito de salubridade ambiental e pode ser empregado no âmbito das políticas públicas, como um condicionante para a priorização de ações necessárias à melhoria das condições de salubridade em aglomerados rurais, visando contribuir com o nível de saúde de suas populações, além de possibilitar uma avaliação da evolução das metas no PNSR e do Plano Municipal de Saneamento Básico. Esse índice pode ser aplicado na sua totalidade ou na avaliação de cada indicador que o compõe.

Os resultados da aplicação do ISA_Rural_nas comunidades estudadas do estado de Goiás indicam que o poder público deve dedicar atenção prioritária para implementar ações que visem a universalização do esgotamento sanitário, seguido da melhoria das condições socioeconômicas, particularmente nas comunidades quilombolas, que apresentaram as piores condições de salubridade ambiental entre as comunidades estudadas.

## Footnotes

a Material suplementar disponível em: https://files.cercomp.ufg.br/weby/up/780/o/Arquivo_complementar_proposicao_e_aplicacao_ISA_Rural.pdf

b Somente o formulário referente aos subindicadores do I_Saúde_obteve mais uma etapa para apresentação e análise das alterações sugeridas pelos especialistas.
